# Structural insights into substrate recognition and translocation of human peroxisomal ABC transporter ALDP

**DOI:** 10.1038/s41392-022-01280-9

**Published:** 2023-02-22

**Authors:** Chao Xiong, Li-Na Jia, Wei-Xi Xiong, Xin-Tong Wu, Liu-Lin Xiong, Ting-Hua Wang, Dong Zhou, Zhen Hong, Zheng Liu, Lin Tang

**Affiliations:** 1 Department of Neurology, State Key Lab of Biotherapy and Cancer Center, West China Hospital, Sichuan University and Collaborative Innovation Center for Biotherapy, 610041 Chengdu, Sichuan China; 2 Institute of Brain Science and Brain-inspired Technology of West China Hospital, Sichuan University, Chengdu, China; 3 Institute of Neurological Disease, State Key Lab of Biotherapy, West China Hospital, Sichuan University, 610041 Chengdu, Sichuan China; 4 School of Life and Health, Kobilka Institute of Innovative Drug Discovery, the Chinese University of Hong Kong (Shenzhen), Shenzhen, China

**Keywords:** Structural biology, Biophysics

## Abstract

Dysfunctions of ATP-binding cassette, subfamily D, member 1 (ABCD1) cause X-linked adrenoleukodystrophy, a rare neurodegenerative disease that affects all human tissues. Residing in the peroxisome membrane, ABCD1 plays a role in the translocation of very long-chain fatty acids for their β-oxidation. Here, the six cryo-electron microscopy structures of ABCD1 in four distinct conformational states were presented. In the transporter dimer, two transmembrane domains form the substrate translocation pathway, and two nucleotide-binding domains form the ATP-binding site that binds and hydrolyzes ATP. The ABCD1 structures provide a starting point for elucidating the substrate recognition and translocation mechanism of ABCD1. Each of the four inward-facing structures of ABCD1 has a vestibule that opens to the cytosol with variable sizes. Hexacosanoic acid (C26:0)-CoA substrate binds to the transmembrane domains (TMDs) and stimulates the ATPase activity of the nucleotide-binding domains (NBDs). W339 from the transmembrane helix 5 (TM5) is essential for binding substrate and stimulating ATP hydrolysis by substrate. ABCD1 has a unique C-terminal coiled-coil domain that negatively modulates the ATPase activity of the NBDs. Furthermore, the structure of ABCD1 in the outward-facing state indicates that ATP molecules pull the two NBDs together and open the TMDs to the peroxisomal lumen for substrate release. The five structures provide a view of the substrate transport cycle and mechanistic implication for disease-causing mutations.

## Introduction

ATP-binding cassette (ABC) transporters are one of the largest families of membrane proteins and mediate the active transport of a wide variety of substrates in different cellular processes, including the uptake of nutrients, movement of lipids and metabolic products across the plasma and intracellular membranes, and multiple drug resistance.^1^ The ABCD subfamily encodes four half-transporters (ABCD1–4), which play important roles in the metabolism of fatty acids and cobalamin and mainly function as a homodimer.^2,3^ ABCD1-3 are frequently expressed in peroxisomes and exhibit distinct but overlapping specificities for different acyl-CoA esters. Among the four members of ABCD family, ABCD1 shares high sequence homology with ABCD2 (Supplementary Fig. 1), and both transporters are suggested to be involved in the transport of long-chain fatty acid-CoA with different substrate specificities. Meanwhile, ABCD3 is involved in the transport of dicarboxylic acid, branched chain acyl-CoA and bile acid intermediates, and di- and tri-hydroxycholestanoyl-CoA. ABCD1 and ABCD2 contain a canonical domain structure of an ABC transporter and a unique C-terminal coiled coil. Mutations of ABCD1 and ABCD3 are associated with two genetic disorders called X-linked adrenoleukodystrophy (X-ALD) and congenital bile acid synthesis defect 5, respectively.^2^

The β-oxidation of very long-chain fatty acids (VLCFAs) is a conserved process in peroxisome, which makes this organelle essential, especially in the brain.^2^ A peroxisomal cycle of β-oxidation is important in the synthesis of polyunsaturated fatty acids, such as DHA. Therefore, in addition to the catabolic function of peroxisomes, peroxisomal ABC transporters have been implicated in the synthesis and degradation of lipids, cell signaling, inflammation, and redox homeostasis. ABCD1, also known as adrenoleukodystrophy protein (ALDP), was identified in 1993 by positioning cloning from patients with adrenoleukodystrophy.^4^ It plays a central role in the transport of VLCFAs. Mutations of ABCD1 commonly lead to the abnormal accumulation of VLCFAs, resulting in X-ALD, a progressive neurological disorder with variable clinical outcomes ranging from adrenal insufficiency to rapidly progressive and fatal cerebral demyelination. X-ALD affects ~1:20,000 males, and more than 900 mutations of ABCD1 have been identified from patients with ALD (https://adrenoleukodystrophy.info/). Approximately 65% of these variants affect protein folding and stability, resulting in a marked reduction in ALDP from the peroxisome membrane.^5^ These mutants cause variable clinical symptoms, including adrenoleukodysfunction, adrenomyeloneurotrophy, and cerebral adrenoleukodystrophy.^6–8^ The early detection of adrenal insufficiency and cerebral ALD through newborn screening can help in the treatment of this disease. Hematopoietic cell transplantation and single-dose gene therapy (Skysona) are some of the effective treatments to halt cerebral demyelination and prevent death when they are administered early, especially before neurologic progression.^9,10^

The disease-causing mutants of ABCD1 contribute to VLCFA accumulation, which in turn affects the stability of adrenal gland, testis, and myelin;^11^ however, the lack of structural information about ABCD1 hampers the understanding of the complex clinical symptoms caused by these mutations. The majority of missense mutations affect protein stability and result in the absence of the protein. A number of mutations (G277R, R280C, and Y296C) result in no defects in the level of ABCD1. In patients with ALD, these mutations are probably involved in stabilizing one of the conformational states of ABCD1. The function of ABCD1 substrate as a straight-chain saturated fatty acid was confirmed during the transfection of human ABCD1 reverse-transcription DNA into X-ALD skin fibroblasts to restore the oxidative activity of VLCFA-β and the VLCFA content in fibroblasts to normal cell levels.^12–14^ Van Roermund et al. proved that ABCD1 is involved in the transport of VLCFA-CoA through the peroxisome membrane to express human ABCD1 in *Saccharomyces cerevisiae*.^15,16^ The ATPase and acyl-CoA thioesterase activity of ABCD1 was recently further characterized through the reconstitution of ABCD1 into liposomes.^17^ The intrinsic acyl-CoA thioesterase activity has also been identified in COMATOSE (CTS), a homolog of human ABCD1 in Arabidopsis thaliana. This finding highlights that VLCFA-CoA is hydrolyzed prior to transport.^18^

Accumulating evidence has shown the important role of ABCD1 under physiological and pathological conditions, especially in the metabolism of VLCFA. However, the lack of structural information on the regulation of ABCD1 by binding to substrate and ATP hinders the understanding of the comprehensive transport of this important peroxisomal molecule under disease conditions. Analysis of the molecular structure of ABCD1 may lead to the discovery of adopted binding sites, which are crucial in developing small-molecule drugs for the treatment of the involved diseases. The atomic structure of ABCD4 was determined by single particle cryo-electron microscopy (EM),^19^ revealing the overall architecture of the ABCD family. However, ABCD1 shares a low sequence identity with ABCD4 (Supplementary Fig. 1) and exhibits different biochemical properties. Meanwhile, ABCD4 functions as a lysosomal cobalamin transporter, and ABCD1 is localized in peroxisome and plays a vital role in the translocation of VLCFAs. At the time of the submission of this manuscript, the other three groups published the cryo-EM studies of ABCD1,^20–22^ revealing how substrate binding stimulates ATP hydrolysis. In the present study, the six molecular structures of ABCD1 in four conformational states were determined by single particle cryo-electron microscopy. These structures show a glimpse of the substrate transport cycle and provide mechanistic implication for disease-causing mutations.

## Results

### Functional characterization of human ABCD1

ABCD1 is a half ATP-binding cassette transporter, and its two subunits dimerize to form a fully functional transporter in the peroxisome. ABCD1 was overexpressed in HEK293GnTl^-^ cells and purified in detergents for further functional characterization. ABCD1 behaved as a dimer on size-exclusion chromatography and ran as a monomer of a 70 kDa protein on SDS-PAGE (Supplementary Fig. 2a, b). The purified ABCD1 and its mutants used for ATPase activity assay were characterized by western blotting using an anti-FLAG antibody, size-exclusion chromatography, and SDS-PAGE analysis (Supplementary Fig. 2d–l).

**Fig. 1:**
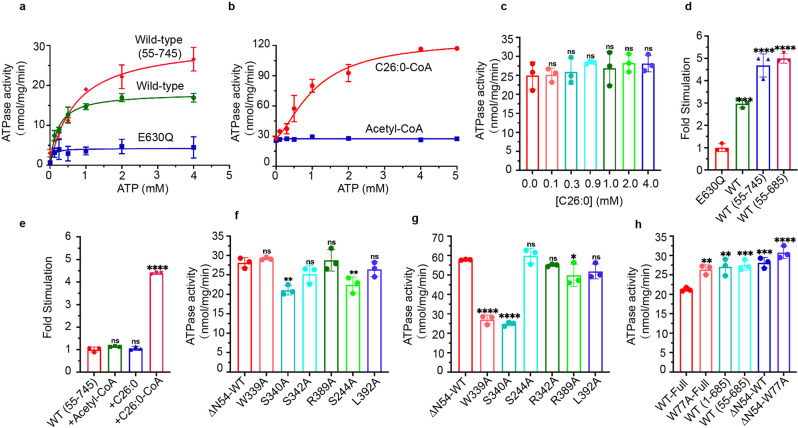
ATPase activity of human ABCD1 stimulated by substrates. **a** ATPase activity of the full-length wild-type, E630Q mutant, and the N-terminal truncated proteins purified in GDN. The full-length wild-type protein showed a K_M_ of 279 ± 18 µM for ATP and a maximal hydrolysis activity of 18 nmol/mg/min. **b** ATPase activity of the truncation mutant devoid of N-terminal 54 residues in the presence of Acyl-CoA at different concentrations. **c** ATPase activity of the truncation mutant devoid of N-terminal 54 residues in the presence of different concentrations of C26:0. **d** Normalized ATPase activity of E630Q mutant protein. The activity of the full-length wild-type protein, truncation mutant devoid of N-terminal 54 residues, and truncation mutant devoid of N-terminal 54 residues and C-terminal 60 residues was simulated. Maximal hydrolysis activity was selected to calculate the stimulated fold. **e** Stimulated ATPase activity of the truncation mutant devoid of N-terminal 54 residues in the presence of C26:0, C26:0-CoA, and acetyl-CoA. The maximal hydrolysis activity was selected to calculate the stimulate fold. **f** ATPase activity of ABCD1 mutants, in which the residues locating in the substrate-binding sites were mutated to alanine. **g** ATPase activity of ABCD1 mutants in the presence of C26:0-CoA. **h** ATPase activity of ABCD1 truncation mutants. The reported errors represent standard deviation. ns no significance^, ✱✱✱✱^*P* < 0.0001, ^✱✱✱^*P* < 0.001, ^✱✱^*P* < 0.01, and ^✱^*P* < 0.05

NADH-coupled ATPase assay was used to measure the K_M_ of ATP and the maximal basal turnover rates of the full-length ABCD1, ABCD1-E630Q mutant, and N-terminal truncation mutant devoid of N-terminal 54 residues, similar to other human ABC transporters^23,24^ (Fig. 1a). The ABCD1-E630Q mutant exhibited one-third of the ATPase activity of the wild-type protein, whereas the N-terminal deletion mutant (ΔN1-54) exhibited a higher ATPase activity (Fig. 1a). Thus, ΔN1-54 ABCD1 was selected to measure the ATPase activity stimulated by the substrate. The K_M_ values for ATP (585 ± 55 µM) and the maximal basal turnover rate (29 ± 1 nmol/mg/min) of ΔN1-54 ABCD1 were higher than those measured for the wild-type ABCD1. The maximal basal turnover rate for the wild-typed ABCD1 is similar to that measured for the ABCD1 in liposome.^25^ In addition, the C-terminus truncation mutant ABCD1 (ΔC 686–745) exhibited higher ATPase activity than the wild-type ABCD1, indicating that the C-terminal region of ABCD1 also plays a negative role in modulating ATPase activity (Fig. 1d).

A remarkable increase in ATPase activity in the presence of hexacosanoic acid (C26:0)-CoA substrate was observed (Fig. 1b), but the addition of either C26:0 or acetyl-CoA showed no stimulation effect (Fig. 1b, c, e). The apparent affinity of ABCD1 for its substrate C26:0-CoA was close to that reported for the ABCD1 measured in vesicles.^25^ Plotting of the ATPase activity as a function of C26:0-CoA concentrations confirmed that C26:0-CoA stimulated ATP hydrolysis by two-fold at saturation concentrations, consistent with substrate-stimulated comatose ATPase activity of 1.8-fold in a plant VLCFA transporter homolog from *Arabidopsis thaliana*. The measured K_M_ value for C26:0-CoA was ~0.3 mM. These findings validated the experimental system and proved that the detergent-purified ABCD1 is reliable and could be used for later structural analysis.

### Structural overview

To elucidate the mechanism of conformational changes during the transport cycle, we collected datasets on the full-length wild-type ABCD1 in the absence of any ligand (apo) and in the presence of C26:0, and the ABCD1-E630Q mutant in the presence of ATP and the ABCD1-E630Q mutant in complex with C26:0-CoA and ATP. Consequently, cryo-EM reconstructions based on these datasets yielded six molecular structures of ABCD1 in four different conformations (Fig. 5, Supplementary Figs. 14–17, and Supplementary Table 1): a wide-open, inward-facing form (inward-facing state 1) at 3.78 Å for the ABCD1-C26:0 complex and 3.33 Å for the ABCD1-E630Q-C26:0-CoA complex (Supplementary Figs. 3 and 6c), an apo form at 3.35 Å (Supplementary Fig. 4), an inward-facing intermediate form (inward-facing state 2) at 3.34 Å (Supplementary Fig. 6d); a nearly closed inward-facing (inward-facing state 3) and outward-facing ABCD1-E630Q (outward-facing state 4) at 3.3 and 2.96 Å in the presence of ATP, respectively (Supplementary Fig. 5d, c).

**Fig. 2:**
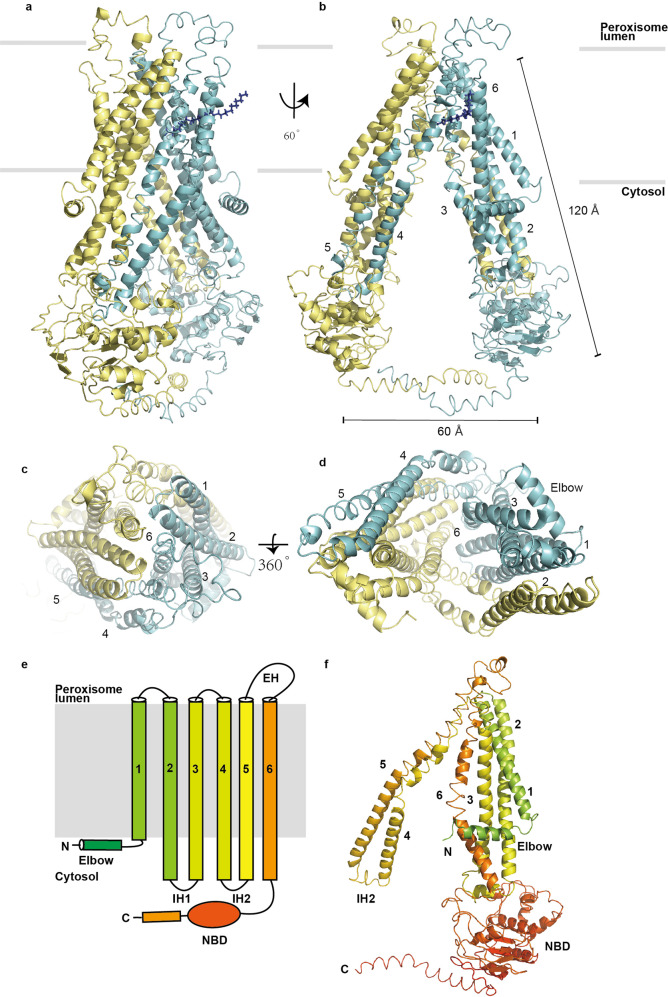
Overall structure of ABCD1 in complex with C26:0 fatty acid. **a**, **b** Cartoon representation of the side view of the overall structure of ABCD1. The two subunits are colored in cyan and yellow. **c**, **d** TMD of ABCD1 viewed from peroxisomal lumen and cytosol. **e**, **f** Domain structures of ABCD1

The EM density map for the wide-open, inward-facing structure (state 1, Figs. 2, 5) was sufficient to build all the transmembrane helices and model the nucleotide-binding domain (NBD) regions (Supplementary Fig. 7a). The region corresponding to the C-terminal coiled-coil helix has a distinct density, which enabled the modeling of their side chains. Three-dimensional (3D) structures consisting of residues 64–724 were built for the ABCD1 in complex with C26:0 and the ABCD1-E630Q in the presence of C26:0-CoA and ATP. The wide-open, inward-facing structure was used to describe the molecular structural features.

The intermediate inward-facing ABCD1 in state 2 had an EM density map with a quality similar to that of ABCD1 in state 1. However, the EM density for the C-terminal coiled coil was missing. Compared with the inward-facing structures (states 1–3), the outward-facing ATP-bound ABCD1 presented considerably better EM densities, which corresponded to the NBD region and cytosolic extensions of the transmembrane helices. Thus, an atomic model of the NBD and the TMD helices was built, except for the loops on the peroxisomal lumen connecting the TMD helices because of their large flexibility and poor EM density maps. In all the above reconstructions, the final structures were refined against the EM maps to obtain good statistics and reliable stereochemical parameters.

Each human ABCD1 subunit contains one 6-transmembrane helical domain, followed by one NBD (Fig. 2e, f) and two subunits assembled into an isosceles triangle structure in the inward-facing state 1 (Fig. 2b). One of the two long sides from the isosceles triangle was the TM bundle 1, which was formed by assembling transmembrane helical structures TM1, TM2, TM3, and TM6 in subunit A with TM4 and TM5 of subunit B. The other long side, also another TM bundle, comprised TM4 and TM5 of subunit A and TM1, TM2, TM3, and TM6 of subunit B. Embedded in lipid bilayer, the two TM bundles cross at a high angle, forming a large vestibule open to the cytosol. The vestibule usually possessed a high-affinity binding site for the substrate, which is an important characteristic of ABC exporters. Furthermore, the IH2 (Fig. 6g) between TM4 and TM5 from one subunit interacts with the NBD of the other via Wan der Waals interactions. The Y296 and E302 of the IH2 of one TM bundle interact with the Y559 of the paring NBD; the ALD variant database showed that the mutations of each of the three residues in human causes X-ALD disease.^26^

Two NBDs located in the cytoplasmic side and 30 Å away from the peroxisomal membrane were bridged by a 60 Å long, curved C-terminal coiled coil. The two NBDs should cross over 40 Å area before coupling tightly together in the presence of ATP and Mg^2+^, and they reasonably influenced the alteration of the conformation in the transmembrane region of ABCD1, from an inward-facing one to an outward-facing structure.

### Substrate recognition

**Fig. 3:**
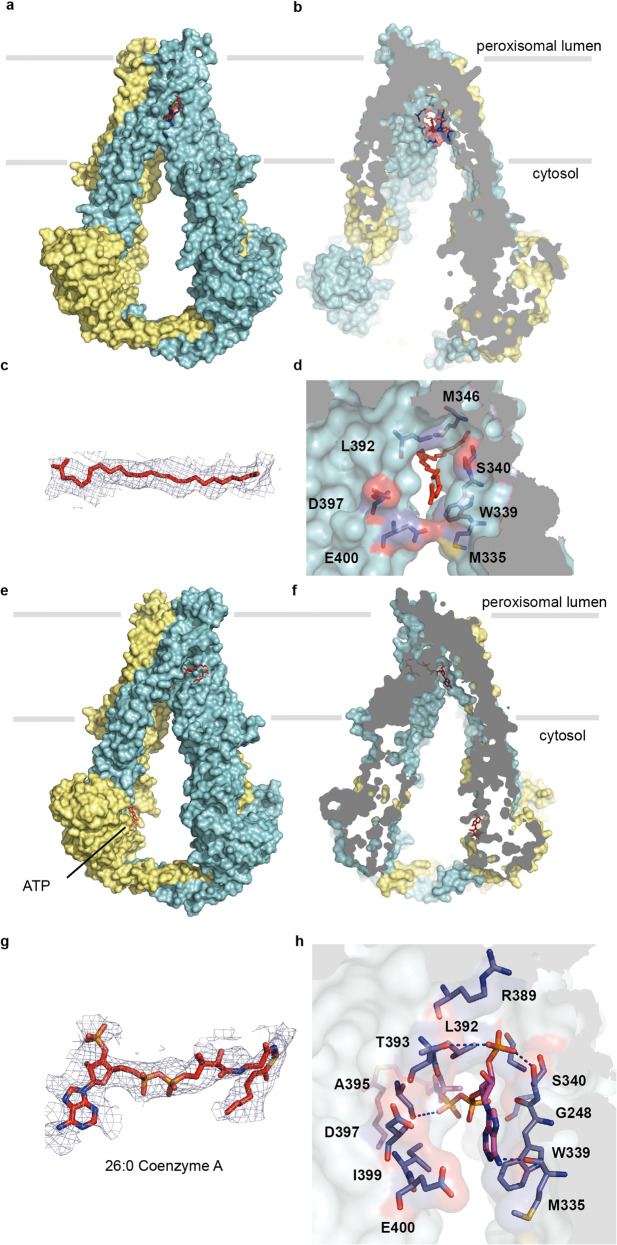
Inward-facing structure of ABCD1 with a cytosolic vestibule that houses the substrate-binding site. **a**, **b** Surface representation of ABCD1 showing that C26:0 binds to a vestibule that penetrates half-way into the lipid bilayer. **c**, **d** Electron density map of C26:0 substrate and zoomed-in view of the substrate in the same orientation as **b**, respectively. **e**, **f** Overall and slice views of ABCD1-E630Q in complex with C26:0-CoA as surface representation, respectively. **g**, **h** EM density of parts of C26:0-CoA and zoomed-in view of the CoA binding site, respectively. The EM density maps in **c** and **g** are displayed at the same sigma level (5σ)

ABCD1 structures were determined in an inward-facing states 1 in the absence of any ligand (apo) and in the presence of C26:0 or C26:0-CoA, in which the C-terminal tail formed a coiled-coil dimer structure, separating the two NBDs. Both the C26:0-bound and C26:0-CoA-bound ABCD1 structures are similar to that of oleoyl-CoA-bound ABCD1 recently reported by Wang R et al. (Supplementary Fig. 13a).^20^ The C26:0 and C26:0-CoA substrate bound to a similar position located at the interface formed by four-helix TM3, TM4, TM5, and TM6 in the TM domain, half away from the cytosol (Fig. 3 and Supplementary Fig. 10). The hydrophilic head group protruded into the vestibule with their tails buried into the fenestration formed by TM3, TM4, TM5, and TM6 (Supplementary Fig. 10a, b). In the case of C26:0, the carboxylic group was located near the inner surface of the vestibule formed by residues E400, D397, W339, and S340 (Fig. 3d), and the tail was situated in the fenestration via hydrophobic interactions. The C26:0-CoA substrate bound to the same position of ABCD1-E630Q as C26:0 in the wild-type ABCD1 structure. Figure 3h shows the interactions between the head group of CoA and the protein, which mainly involved hydrophobic and hydrogen bond interactions with the surrounding residues. The W339 and M335 from TM5 packed with the adenine moiety from one side via hydrophobic interactions, whereas T393, D397, and E400 made close contact from the other side (Fig. 3h and Supplementary Fig. 7d). The pantetheine arm extended into the fenestration, encircled by A395, L392, S340, and G248. The fatty acid tails of C26:0-CoA was flexible given their less defined density. Among the ligand-interacting residues at the substrate-binding site, W339A and S340A exhibited the most profound effect on the ATPase activity of ABCD1 stimulated by C26:0-CoA (Fig. 1g), indicating that both residues probably participate in the coupling between substrate and ATP hydrolysis. The MD simulations of ABCD1-ATP-C26:0-CoA complex showed that the protein, ligand and ATP molecules underwent fluctuations and finally rested at around 3.5, 2.0, and 4.0 Å, respectively (Supplementary Fig. 11a, b). In the initial structure, the phosphate group connected to the ribose was hydrogen-bonded only to S340. After the simulation, it formed a strong salt bridge with K336 in addition to the hydrogen bond to S340. The adenine group was now surrounded by more hydrophobic residues, including W339, F252A, A396, I399, and M403. Although the core region of the ligand molecule exhibited small deviation from the initial structure, the whole ligand molecule had a considerably larger RMSD from the initial structure, which was around 9.5 Å. This finding resulted from the large magnitude of alternation in the position of the aliphatic chain. At this point, the aliphatic chain was surrounded by lipid molecules, and showed large conformational fluctuation during the simulation (Supplementary Fig. 11c–e). After the 100 ns simulation, the ATP molecule resides nearly the same pocket as it was in the initial structure (Supplementary Fig. 11f, g).

In the absence of any ligand, the substrate-binding site was occupied by an endogenous lipid based on the EM density map (Supplementary Fig. 12c). The density for one acyl chain of the lipid was visible, but it was not sufficient for us to place the whole lipid molecule into the map. The lipid head group was located near the inner surface of the vestibule, distinct from the carboxylic group of C26:0 (Supplementary Fig. 12a, b, d). Its acyl tail was also inserted into the fenestration formed by TM3, TM4, TM5, and TM6, similar to the tail of C26:0 and C26:0-CoA (Supplementary Fig. 12e, f).

A unique feature of ABCD1 in state 1 in this study was that the distance between the two NBDs approached 50–60 Å, which was beyond the anticipated value before this test. The distance between the ATP-combining site (Walker A motif) and the signature motif was notably 38 Å (Fig. 5a), which agrees with a previous report, and the distance of the two NBDs of CFTR under closed state uniquely reached 45 Å.^27^ The separation of the two NBDs with a large distance (>40 Å) is questionable at physiological levels of ATP.^28^ Meanwhile, the presence of the coiled-coil helix in the inward-facing state 1 probably configured the protein to be ready for binding the long-chain fatty acid substrate.

Among other ABC transport proteins, such as MRP1 structure, the distance between two NBDs of ATP-binding sites was wide, as reported previously, attaining a mean level of 35 Å.^24^ In the presence of substrate, the Walker A/B motifs and the signature motif consisting of the two ATP-binding sites of MRP1 were uncommonly separated in a non-parallel manner (Supplementary Fig. 13c, d). In the present study, the parallel opposite locations of the two NBDs of ATP-binding sites may be attributed to the C-tail of ABCD1, causing the two NBDs of the ATP-binding site to line up in a relatively parallel location.

Structural comparison of the two NBDs in the C26:0-bound structure revealed that the end of the C-terminal coiled coil from one subunit interacted differently with the other NBD. In one NBD, the E722 from the coil helix was positioned closely to the P508 from the Walker A motif (Supplementary Fig. 7b, c), whereas the E722 in the other subunit moved away from residue P508. The sequence of the Walker A motif (GPNGCGKSS, Supplementary Figs. 1 and 9g) was conserved among ABCD1, ABCD2, and ABCD3 but different from that of ABCD4. Furthermore, an ATP molecule that binds to the Walker A motif of the same NBD was defined in the C26:0-CoA bound structure (Supplementary Fig. 9b), in which E722 made close contact with P508. The ATPase activity of P508A mutant was lower than that of wild-type ABCD1 (Supplementary Fig. 2c), indicating that P508 plays a role in regulating ATP hydrolysis. Furthermore, we measured the transporter ATPase activity using the C-terminus truncation mutant ABCD1 (ΔC 686–745) to confirm the role of the C-terminal coiled coil. This mutant increased the basal ATPase activity by almost one-fold compared with that of the wild-type ABCD1 (Fig. 1h).

### Inward-facing conformational intermediate of ABCD1

A general feature of the ABC family is that binding of substrates to the TMDs primes the transporter for ATP hydrolysis. In this study, experiments were conducted to elucidate the role of C26:0 and C26:0-CoA in promoting ABCD1 ATPase. C26:0-CoA, but not C26:0, significantly elevated the vitality of ABCD1 ATPase activity by approximately two-fold (Fig. 1b). Furthermore, a test referring to other methods was performed, in which hydrolysis of ATPase was set up in typical biochemistry experiments by using the purified ABCD1-E630Q. This mutant reduced the ATPase activity to one half of the wild-type ABCD1. Therefore, on the basis of these findings, the structural changes under the condition of ABCD1 with the addition of substrate compounds were investigated.

The 3D reconstructions of the complex of ABCD1-E630Q mutant with C26:0-CoA and ATP yielded an additional inward-facing state 2 (Fig. 5c and Supplementary Fig. 8), in which the density of the C-terminal coiled coil was missing. Structural comparison between ABCD1 structures in states 1 and 2 showed that the C-terminal coiled-coil dimer could sterically clash with the two NBDs in inward-facing state 2 (Supplementary Fig. 8d). Under the special state, TM4 moved towards TM3 and TM6 (Supplementary Fig. 10c), closing the fenestration that extended from the TM4–TM3 interface to the substrate-binding site near the W339 residue (Supplementary Fig. 8b). Consequently, the side chains of W339 from TM5 and R401 and K407 from TM6 closed the open court by forming cation-π interactions, without the possibility of combining with the CoA part into the position near W339 (Supplementary Fig. 8b). The undergoing movement of the TM bundles formed a small angle between the two TMDs with a slit surrounded by residues N256, R259, T416, R411, and F252 (Supplementary Fig. 8c). Its position was close to the N-terminal region of the elbow, which laid outside the TM6 and TM3 helices. However, the EM density for the N-terminal 54 residues preceding the elbow could not be defined. Several other reports of ABCD1 structures did not show the structure of the N-terminal region.^20–22^ Previous studies suggested that the mutation of residues in the elbow affected the folding of ABCD1, resulting in low protein levels in body tissues.^29^ The tight interaction between the elbow and TM6 could retain the elbow attachment onto TM6 during the movement of the inner parts of the TM domains during the transport cycle. The W77A mutant resulted in higher ATPase activity of ABCD1 than the wild-type ABCD1 (Fig. 1h), indicating that the elbow negatively regulates the ATPase activity of ABCD1. Therefore, the preceding region of N-terminal 54 residues probably affects the opening of the TM bundles and thus prevents the exposure of the substrate-binding site. In this kind of conformation, two NBDs moved closer to each other, resulting in the disruption of the dimerization of the coiled coil in C-tail. One ATP molecule that bound to the Walker A motif in the NBD of one subunit was also identified (Supplementary Fig. 9c). Moreover, the deletion of N-terminal 54 amino acids significantly increased the ATPase activity of ABCD1 with and without the addition of C26:0-CoA compared with the full-length protein (Fig. 1a, b). These results suggested that the N-terminal region preceding the elbow has an inhibitory role against ABCD1, probably regulating the opening of the two bundles for exposure of the substrate-binding site.

### ATP binding drives inward-to-outward conformational transitions

**Fig. 4:**
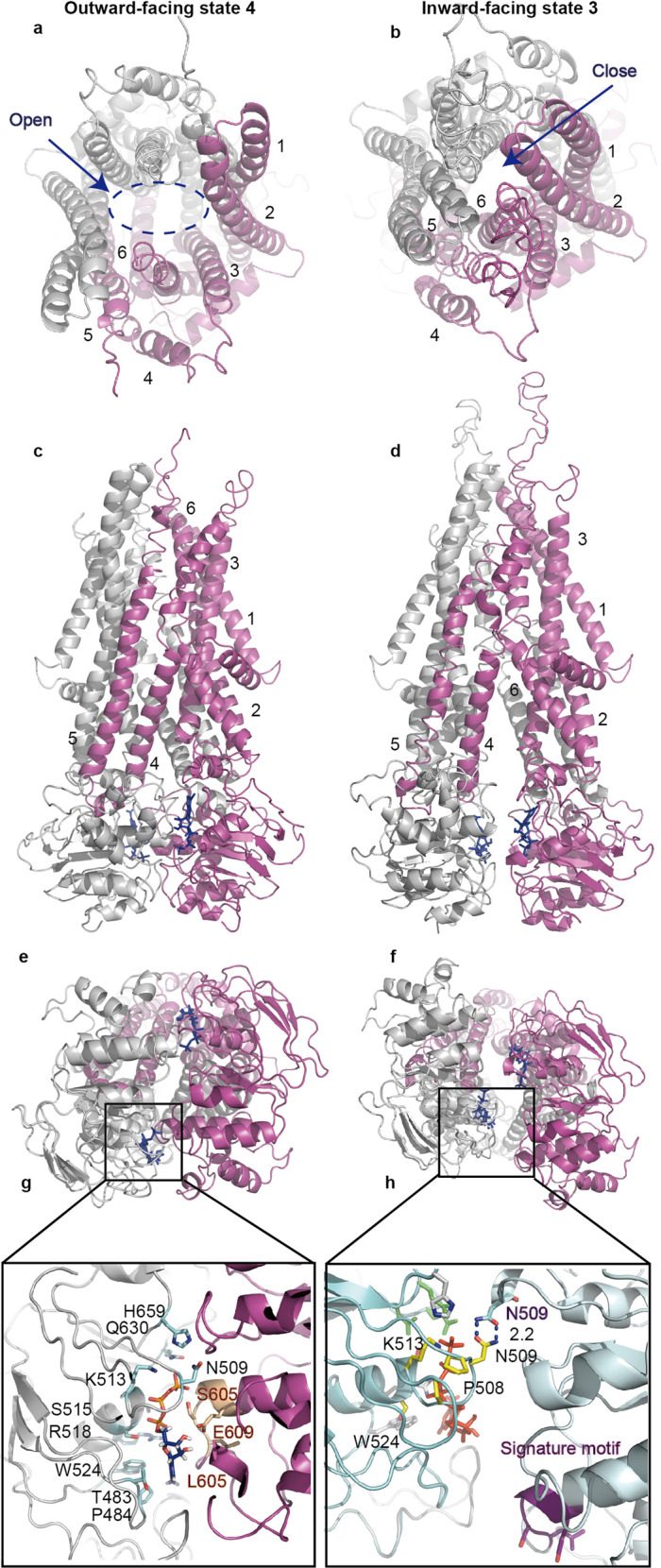
ATP-bound inward-facing and outward-facing structures of ABCD1. **a**, **c**, **e**, **d** Cartoon structures representing ABCD1 in complex with ATP in outward-facing state. **a**, **e** View from the peroxisome lumen side and cytosol; **c** side view. **g** Zoomed-in view of the ATP-binding site. **b**, **d**, **f**, **h** Cartoon representations of ABCD1 in the complex of ATP in inward-facing state, viewed from a similar orientation as the outward-facing structure on the left

ABCD1 is a kind of half transporter needed for the dimerization of two subunits to form functional transporter. In the inward-facing state 1, the C-tails of ABCD1 formed dimers, thereby causing the alignment of the ATP-binding sites in parallel. The ABCD1-E630Q structure was determined in the presence of ATP without substrate to confirm ATP binding and define its role in ABCD1 activity, thus allowing the identification of two bound ATP molecules in the NBD dimer interface. Unexpectedly, a new inward-facing structure (inward-facing state 3) was determined, in which two ATP molecules bound to the NBDs (Fig. 4b, d, f, h and Supplementary Fig. 9d). However, the two NBDs were twisted, with the complete separation of the Walker A and the signature motifs of the ATP-binding sites. ATP molecules only bound to the Walker A motif of either NBD, and no EM density corresponding to Mg^2+^ could be identified. Without the interaction with the signature motif, the ATP molecules could not be hydrolyzed normally. Structural comparison of the ATP-binding sites in ABCD1 between states 2 and 3 showed that N509 switched from the interaction with H659 in state 2 to N509 from the paired subunit in state 3 (Supplementary Fig. 9c, d). The N509I mutant has been reported to be associated with ALD.^30^ Meanwhile, in ABCC1 and ABCD4, the corresponding residue changes were attributed to valine and threonine, respectively (Supplementary Fig. 9g). The conservation of N509 residue in ABCD1-3 members suggested that it may play an important role in the dimerization of the two NBDs during the translocation of VLCFAs. The binding of ATP molecules to three states of ABCD1 without hydrolysis suggested that they modulate the activity of the protein by regulating key residues, such as H659, N509, and P508.

The outward-facing ATP-bound ABCD1 structure was determined at a resolution of 3.0 Å, which is equivalent to that of ATP-bound outward open structure of ABCD1 recently reported by other groups (Fig. 4a, c, e, g and Supplementary Fig. 13b).^20,21^ In the ATP-bound outward state, the interaction between both N509s was disrupted, and each N509 formed two hydrogen-bonding interactions with the carbonyl of S633 and the main chain nitrogen of V635 to stabilize the canonical ATP-binding site (Supplementary Fig. 9e). These hydrogen-bonding interactions were identified in the observation, and they implied that the normal function of ABCD1 must be maintained because mutation of the three residues could lead to diseases. The adenine moiety of ATP molecule was sandwiched between the P484 and W524 of one NBD and L605 from the other NBD (Fig. 4g) The phosphate group of ATP molecule formed electrostatic interactions with the Walker motif, the Q-loop of one NBD, and the signature motif of the other NBD. ATP molecule acted as a glue to staple the two NBDs together, thereby bringing two bundles of TMDs together and forming new interactions between one NBD and the IH1 and IH2 of the paired TM bundle (Supplementary Fig. 9f). Mg^2+^ cation was identified to coordinate with residue Q544, D629, S514, and E630 (Supplementary Fig. 9e). Therefore, these interactions possibly coupled the conformational changes induced by the binding of ATP to the TMD and drove the transition of inward-facing state to outward-facing state.

**Fig. 5:**
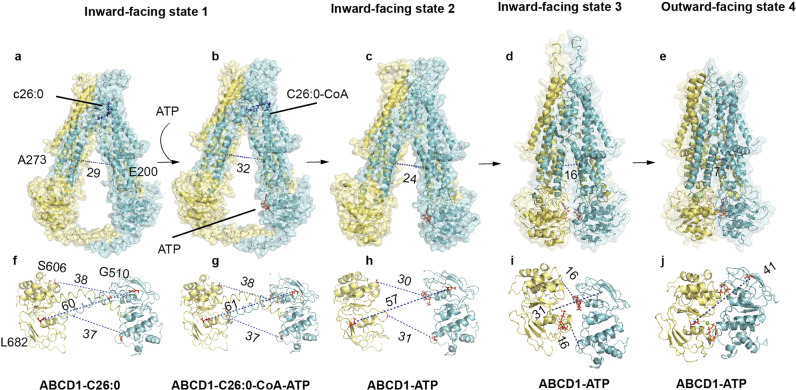
Three inward-facing states and one outward-facing state of ABCD1. **a**, **b** Surface representation of ABCD1 in inward-facing state 1, characterized by the presence of C-terminal coil. **c** Structure of ABCD1 in inward-facing state 2, with a shortened distance of two NBDs compared with that in state 1. **d** ABCD1 with narrower vestibule opening to the cytosol when captured in inward-facing state 3. **e** ATP stapling the two NBDs and the outer segments of the TMD pealed open to the peroxisome lumen. **f**–**j** Measurement of the distance between the two NBDs based on the different states of ABCD1 above

The conformational changes in ABCD1 during substrate release could be extrapolated by comparing ATP-bound inward-facing and outward-facing structures. The closure of NBDs by the binding of ATP brought the two halves together, as indicated by the shortened distance between the cytoplasmic parts of TM3 and TM4 from 16 Å in the inward-facing state to 7 Å in the outward conformation (Fig. 5d, e). The cytoplasmic part of TM5, which is directly linked to TM4 through the inner IH2 and tightly makes hydrophobic contacts with TM3, moved towards the opposite half and closed the deep vestibule that was open to the cytosol in the inward-facing conformation (Fig. 4c, d). The inner helix of TM2, which connects to TM3 through IH1 and tightly contacts with TM3, also moved along with TM3 to help seal the vestibule. The elbow embraced the surface of TM3 and TM6, similar to a string, although tight hydrophobic interactions may play roles in regulating the conformational change in TM3 and TM6. The extracellular ends of the TM helices rotated outwardly and opened the transmembrane vestibule to the peroxisomal lumen. The outward-facing structure of ABCD1 showed a remarkably large vestibule, which sealed the deep cytoplasmic side with electrostatic interactions between charged residues (Supplementary Fig. 10g). These residues at the bottom of the vestibule included K276 and R280 from TM4 and Q195 and D200 from TM3 (Supplementary Fig. 10g). The inner surface of the vestibule was decorated with a number of charged residues, which may exclude the binding of the hydrophobic tails of the long-chain lipid and promote its release into the peroxisome. Similar to the outward-facing structure of ABCC1, no substrate could be identified in the vestibule, which may not possess a binding site with sufficiently high affinity for substrate capture. This outward-facing structure provided a glimpse of the substrate-release state of ABCD1.

## Discussion

### Conformational changes during the translocation process of ABCD1

In this study, three inward-facing conformations and one outward-facing conformation were captured (Fig. 5 and Movie S1). The distance between S606 of the signature motif of one NBD and G510 of the Walker A motif of the other NBD in all the above states was measured, except for the outward-facing conformation, in which both residues came together to bind the ATP molecule. The E682 at the end of the final β-strand of a typical NBD was another residue selected for the measurement of the relative position of the two NBDs in different states. For analysis of the conformational changes in the TMD, the distance between D200 of TM3 and A273 of TM4 was measured, both of which moved close to a distance of 7 Å in the outward-facing state upon binding of ATP (Fig. 5e).

Comparison of these structures showed that the two ATP-binding sites were 38 Å apart, while TM3 and TM4 were 29 Å apart in state 1 (Fig. 5a, f). In this state, the C-terminal of the coiled coil of one NBD closely attached to the Walker A motif of the other NBD. Thus, the N509 at the Walker A motif could stabilize the phosphate group of the ATP molecule (Supplementary Fig. 9b). Upon binding of substrate and ATP, the two NBDs moved towards each other along the axis of the C-terminal coiled coil, and the distances between the two separated ATP-binding sites were shortened by 8 Å in state 2 (Fig. 5c, h). The C-terminal coiled coil was missing in this state; otherwise, it could block the movement of the two NBDs. Without the interaction with the C-terminal coiled coil, the N509 of the Walker A motif interacted with the phosphate group of ATP molecule (Supplementary Fig. 9b). Furthermore, the two bundles of TMDs moved closer, with a shortened distance of ~6 Å (Fig. 5c). In state 3, the two NBDs were very close, with the new interaction of N509 from both subunits (Fig. 5i and 4h). However, the ATP-binding sites were completely separated, with a distance of 16 Å between S606 from one NBD and N509 from the other (Fig. 5i). In state 3, ATP bound to the Walker A motif, with its phosphate group stabilized by K513, Q630, D629, and Q544 (Supplementary Fig. 9d). Although ATP molecules could bind to one NBD independently, they could not be hydrolyzed without the interaction of the signature motif from the other subunit. Therefore, the binding of one ATP molecule to the Walker A motif of one NBD probably played a role in pulling the signature motif from the other subunit. The distance between TM3 and TM4 was shortened further by ~8 Å compared with their distance in state 2 (Fig. 5c, d). However, the inner halves of the transmembrane segments did not seal the vestibule yet at the cytosolic side in this state. Until the two NBDs were stapled together (Fig. 5e, j), conformational changes occurred at the NBD–TMD, and the inner halves of the transmembrane segments propagated to the outer halves of TMDs and opened the vestibule to the peroxisomal lumen, subsequently releasing the substrate into the peroxisome. The hydrolysis of ATP molecules reset the transport cycle. In conclusion, the structural analysis indicated that binding of the substrate and ATP molecule increased the transport activity of ABCD1 efficiently because the two NBDs were separated by a large distance of the C-terminal coiled coil. The binding of ATP released the brake acted by the C-terminal coiled coil by influencing the Walker A motif, which was coupled to the C-terminal of the coiled coil of another subunit.

### Structural basis of disease-causing mutations

**Fig. 6:**
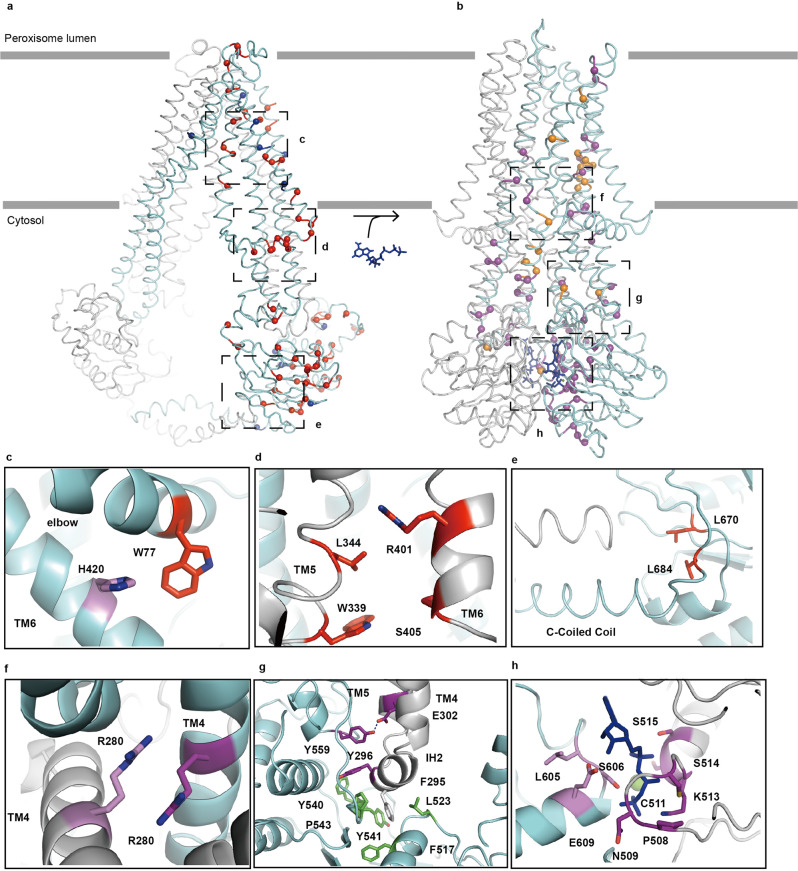
Mapping of the locations of disease-causing mutations by using the inward-facing and outward-facing structures of ABCD1. **a** Red balls represent the deleterious mutations causing the reduced expression of ABCD1 in human tissues; they were selected because they mostly form interactions in the inward-facing structure. Blue balls indicate the mutations that cause no effect on the level of proteins in human tissues but result in normal expression levels of proteins with functional defects. **b** Location of mutations on the structure of ABCD1 in outward-facing state. Purple balls represent the deleterious mutants, and orange balls indicate the mutations that affect the function of ABCD1 but cause no folding problem. **c**–**h** Roles of representative residues by 3D structural analysis

ABCD1 is the gene with the most mutated loci found in the ABC family, and most of such mutations are pathogenic. The database (https://adrenoleukodystrophy.info/) or previous literature was analyzed to map these mutations, where the red-labeled mutations cause protein misfolding and degradation. The results showed that most of the mutation sites were evenly distributed along the full-length protein (Fig. 6). A total of 1226 mutations have been reported, with 826 causing diseases. Most of these mutations influence the folding and stability of the protein, resulting in reduced protein levels in patients, with approximately 30 mutations of non-functional protein, although they reach normal levels in human tissues.^31,32^ Considering the large conformational changes during the transport process, the mutation sites probably affect the inward-facing and outward-facing conformations differently. Thus, their positions were mapped separately onto the inward-facing and outward-facing structures on the basis of their roles in maintaining one of the states. The inner helixes of TM3 and TM4 interact only in the outward-facing state. Therefore, the mutations on this region were mapped in the outward-facing conformation. Several sites of residues interacted during the entire transport cycle. Most of the interacting residues were located at key positions of ABCD1, such as the TMD intersection, NBD-TM interface, ATP-binding site, and substrate-binding site. Mutations of these positions are associated with diseases, and in this study, they were labeled as red spheres in the inward-facing conformation or purple spheres in the outward-facing conformation. By contrast, mutations without effects on protein expression levels were observed. However, these proteins were loss-of-function proteins, and these mutations were separately labeled with blue and orange colors in the inward-facing and outward-facing structures, respectively (Fig. 6a, b).

The number of mutations was incredibly larger than the total number of amino acid residues. Among the 826 missense mutations, 218 mutations affect the folding or function of ABCD1, and and they were mapped separately on the inward-facing and outward-facing structures. Therefore, understanding the action of each residue and cluster of residues in maintaining a certain conformation or triggering the alteration of conformational states is valuable for the development of correcting molecules and potentiators for the treatment of X-ALD. The United States Food and Drug Administration-approved drug ivacaftor for the treatment of cystic fibrosis increases the open probability of wild-type and mutant CFTRs.^33,34^ A similar drug development strategy may be possible to develop activators for mutant ABCD1 given that 30 mutants influence the activity of ABCD1.

Structural analysis of the inward-facing and outward-facing conformations of ABCD1 revealed a number of clusters of interacting residues, whose mutations could cause diseases. Figure 6c–e presents examples of the interacting residues on the inward-facing state of ABCD1. The W77 on the elbow interacted with the H420 from TM6 (Fig. 6c), and the mutation of either of them resulted in the low expression of ABCD1.^35,36^ The enhancement of the ATPase activity of ABCD1 by the N-terminal deletion and W77A mutant indicated that the elbow and its preceding N-terminal region may have a role in regulating the activity of ABCD1 via direct contact with TM6. Meanwhile, L684 was positioned at the very beginning of the C-terminal coiled coil (Fig. 6e), and its mutation to proline may affect the correct orientation of the coiled-coil helix, decoupling its interaction with the Walker A motif of the NBDs. Furthermore, mutations to various residues have been reported on positions W339 and R401,^32,37–40^ which were situated at the substrate-binding site (Fig. 6d). In addition, a large number of interactions appeared in the outward-facing conformation of ABCD1. The inner helix of TM4 on the separated TM bundles moved close to each other in the outward-facing conformation only, and the R280 on both helixes formed a salt bridge interaction at the very bottom of the outward-opening vestibule (Fig. 6f). The mutation of R280 to cystine may lock the protein in an outward-facing conformation.^32,41^ Further structural studies of R280C are needed to clarify its influence on the function of the protein. Many cases have been reported for the S606 and G512 positions from signature and Walker A motifs, respectively,^40,42,43^ which interact with ATP molecules in the outward-facing conformation (Fig. 6h). A large number of mutations occurred at the ATP-binding site, which may affect the activity of ABCD1. Lastly, the pathogenic mutation of Y559 (Fig. 6g)^26^ may affect the interaction of the NBD of one subunit with the IH2 of the other one.

In summary, a series of conformations of ABCD1 from the inward-facing state 1 was presented for the binding of substrates to the outward-facing state for substrate release (Fig. 6 and Movie S1). The substrate and ATP increased the activity of ABCD1 through TMDs and NBDs. The C-terminal coiled coil, a unique feature of ABCD1, regulated the activity of the protein by direct contact with the Walker A motif of the other NBD. Furthermore, a map of a large number of mutations on the inward-facing and outward-facing structures was developed. These structures not only provide a glimpse of conformational changes during the transport cycle but also offer a basis to understand human X-ALD and facilitate drug development.

## Materials and methods

### Cell culture

HEK293S GnTl^-^ suspension cells were cultured in Freestyle 293 medium (GIBCO) at 37 °C and supplemented with 8% CO_2_, 2% fetal bovine serum, and 80% humidity.

### Expression and purification of human ABCD1

Full-length human ABCD1 gene was synthesized by General Biosystems. The human ABCD1-E630Q was introduced using a standard two-step polymerase chain reaction. The wild-type and mutant genes were cloned into a pcDNA3.1 vector with an N-terminal FLAG tag (DYKDDDDK). HEK293S GnTl^-^ cells were transiently transfected with the expression plasmid and polyethylenimines (PEIs) (Polysciences) when the cell density reached 3 × 10^6^ cells per mL. For 3 L of HEK293S GnTl^-^ cell culture, 5 mg plasmids were premixed with 10 mg PEIs in 300 mL fresh medium for 5 min. Then, the mixture was added to the 3 L cell culture. The transfected cells were incubated at 37 °C for 72 h and harvested.

For purification, after centrifugation was conducted at 4000 rpm for 30 min, the cell pellets were resuspended and solubilized for 3 h at 4 °C in a lysis buffer containing 150 mM NaCl, 25 mM Tris (pH 8.0), 2 mM MgCl_2_, 20% (v/v) glycerol, 1% (w/v) n-dodecyl-β-D-maltopyranoside (DDM), and 0.1% (w/v) cholesteryl hemisuccinate (CHS) supplemented with protease inhibitors (1 µg/mL leupetin, 1 mM benzamidine, 100 µg/mL soy trypsin inhibitor, 1 mg/mL pepstatin, 1 µg/mL aprotinin, and 1 mM phenylmethylsulfonyl fluoride). After centrifugation was performed at 40,000 rpm for 1 h, the supernatant was applied to anti-FLAG M2 affinity gel (Sigma–Aldrich) at 4 °C for 2 h. The resin was packed into a column and washed with wash buffer containing 150 mM NaCl, 25 mM Tris (pH 8.0), 2 mM MgCl_2_, 5% (v/v) glycerol, 0.02% (w/v) DDM (Anatrace), and 0.002% CHS (Anatrace). The protein was eluted with the wash buffer supplemented with 0.2 mg/mL FLAG peptide. The flow through was collected and concentrated by a 100 kDa Centricon (Milipore) and further purified by Superose 6 size-exclusion column (GE Healthcare) equilibrated in 150 mM NaCl, 25 mM Tris (pH 8.0), 2 mM MgCl_2_, and 0.04% GDN. Peak fractions were pooled and concentrated for ATPase assays or cryo-EM experiments.

### Western blot analysis

Approximately 2 μg of ABCD1 and ABCD1 mutant proteins was denatured and applied to 10% SDS-PAGE and then transferred to polyvinylidene difluoride membranes (Millipore). The membranes were blocked with 5% skim milk powder in PBST (phosphate-buffered saline with 0.1% Tween 20) at room temperature (RT) for 2 h. Then, the membranes were incubated with primary rabbit anti-ABCD1 monoclonal antibodies (1:4000 dilution in PBST; Proteintech;18138-1-AP) over night at 4 °C. On the next day, the membranes were incubated with horseradish peroxidase-conjugated goat anti-rabbit IgG (H + L, 1:3000 dilution in PBST; Proteintech; SA00001-2) at RT for 2 h. The membranes were washed with PBST three times, 10 min every time. Then, they were developed with Clarity Western ECL substrate (170-5060, BIO-RAD) on a VILBER BIO IMAGER.

### ATPase activity assay

The ATPase activity of human ABCD1 was determined using enzyme-coupled reactions that oxidized NADH after ATP hydrolysis to ADP.^44^ The reaction buffer contained 0.04% GDN, 150 mM KCl, 50 mM HEPES (pH 7.5), 10 mM MgCl_2_, 2 mM dithiothreitol, 60 µg/mL pyruvate kinase, 32 µg/mL lactate dehydrogenase, 4 mM phosphoenolpyruvate, and 150 µM NADH, and a final concentration of 0.8 µM protein was added. Reactions were started by the addition of ATP and incubated at 37 °C. The NADH consumption was calculated by detecting the fluorescence at λ_ex_ = 340 nm and λ_em_ = 445 nm with the use of a CLARIOstar microplate reader (BMG). The rates of ATP hydrolysis were determined by the consumption of NADH, and the fluorescence loss of NADH was calculated by the standards of NADH. V_max_ and K_M_ were calculated using GraphPad Prism by fitting the Michaelis–Menten equation. Then, the substrates were added to the reaction mixture. The mixture was pre-incubated for 10 min before adding 4 mM ATP to initiate the reaction.

### Molecular dynamics simulation methods

The simulation of ABCD1 was based on the cryo-EM structure of ABCD1-E630Q-C26:0-CoA. Two magnesium cations were added to the original complex to balance the negative charges of the phosphate groups. One of the magnesium cations was placed next to the α- and β-phosphate groups in the ATP molecule, and the other was placed next to the diphosphate group in the ligand molecule. Lipid molecules, which comprising of POPC: POPE: POPG at a molar ratio of 3:1:1, were added using the PACKMOL-Memgen program in the AmberTools 22 package. Meanwhile, 96 potassium cations and 80168 water molecules were added to solvate the extramembrane region of the protein. The whole system was then neutralized by 7 sodium cations.

AMBER19SB, GAFF2, and TIP3P force field parameters were assigned to the protein, ligand and water molecules. The force field parameters for ATP were downloaded from http://amber.manchester.ac.uk/. The whole system was energy-minimized for 5000 steps of the steepest descent and 5000 steps of conjugate gradient minimization algorithms, during which a strong restraining potential (100 kcal⋅mol^−1^·Å^*−*2^) was applied to the protein, ATP and the ligand molecules. In the second step, the restraint was removed, and the whole system was minimized for 20,000 steps. The fully relaxed system was heated up to 300 K in 1 ns with a weak restraining potential (10 kcal⋅mol^−1^·Å^*−*2^) applied to the protein, ATP, and ligand molecules. The total volume was fixed during heating. Then, the volume was relaxed in a 2 ns NPT simulation using the Monte Carlo barostat. In the subsequent production simulation, the whole system was further relaxed. The nonbonded interaction was truncated at 9 Å, and the long-range electrostatic interaction was calculated using a particle mesh Ewald. The temperature was regulated using Langevin dynamics with a collision frequency of 1.0 ps^−1^.

### Cryo-EM sample preparation and data collection

Purified ABCD1 was concentrated to 3 mg/mL, followed by frozen-sample preparation. In preparing the ABCD1-ATP sample, 1 mM of ATP, and 2 mM of MgCl2 were added in the buffer used for size-exclusion chromatography. For the ABCD1-C26:0 complex, 100 µM of C26:0 was added in the buffer used for running an anti-Flag affinity column and kept at the same concentration in the buffer used in the following purification steps. In obtaining the ABCD1-C26:0-CoA-ATP complex, we added 1 mM of ATP, and 2 mM of MgCl2 to the running buffer used for anti-Flag affinity purification and kept at the same concentration in all the following steps. In addition, 100 µM of C26:0-CoA was added to the ATP-bound ABCD1 complex after size-exclusion chromatography and incubated for 30 min, and then the sample was concentrated for cryo-EM grid preparation. The 3 µL protein sample was applied to glow-discharged holey carbon grids (Quantifoil Au R1.2/1.3, 300 mesh). After incubation on the grids at 4 °C under 100% humidity, the grids were then plunged frozen in liquid ethane by using a Vitrobot (Thermo Fisher). Cryo-EM datasets were acquired on a Titan Krios microscope operated at 300 kV with a K3 Summit direct electron detector. Images were recorded with serial EM,^45^ with magnification at 105 K and a pixel size of 0.83 Å. The defocus range was from −1.5 to −2.5 µm. Each micrograph was dose fractionated to 50 frames under a dose rate of 1.33 e^−^.

### Cryo-EM data processing

For the ABCD1-ATP sample, 4152 micrographs were obtained after careful filtering by patch motion correction and patch CTF (Cryosparc)^46^ from 4290 movies. A total of 2000 particles were manually picked, followed by two runs of 2D sorting for automatic particle selection template. Exactly 14,993,572 particles were selected based on the 2D sorting template, and 636,284 particles were obtained for the next step calculation after several runs of 2D sorting to remove bad particles. The initial models in this study were generated by Cryosparc, in which 2,188,534 particles were filtered by heterogeneous and non-uniform refinements. Then, two conformations were obtained at the resolutions of 3.30 and 2.96 Å (Supplementary Fig. 4 and Table 1).

For the ABCD1-C26:0 dataset, 8757 movies were obtained first, and 8324 micrographs were selected after careful filtering by patch motion correction and patch CTF (Cryosparc). This step was followed by manual selection of 2000 particles for 2D classification twice to generate an automatic particle selection template. After bad particles were removed after several runs of 2D classification of 4,224,743 particles, a total of 731,411 particles were selected for the next calculation. Subsequently, two of the initial models (505,844 particles) that were generated by Cryosparc were filtered by heterogeneous and non-uniform refinements, and one conformation was obtained with resolutions of 3.78 Å (Supplementary Fig. 3 and Table 1).

In the absence of any ligand (apo form), 4545 movies were collected on the full-length ABCD1, and 4350 micrographs were selected for particle picking after the careful filtering by patch motion correction and patch CTF. A total of 1,521,885 particles were selected for the following 2D classification, and after several runs of 2D classification steps, 609,111 particles were selected for the next calculation. The initial models were generated by Cryosparc and were further filtered by heterogeneous and non-uniform refinements, and one inward-facing conformations was obtained with a resolution of 3.35 Å (Supplementary Fig. 4 and Table 1).

For the ABCD1-C26:0-CoA-ATP sample, 10,159 movies were collected before the delivery to patch motion correction and patch CTF estimation (Cryosparc), and 9622 micrographs were selected carefully. A total of 2000 particles were manually selected, followed by two runs of 2D classification for the generation of automatic particle selection template. On the basis of the template, a total of 6,835,392 particles were obtained and filtered two times via 2D classification for the removal of bad particles. A total of 63,010,722 particles were selected for the next steps. The initial models generated by Cryosparc were filtered by heterogeneous and non-uniform refinements, and two representative inward-facing conformations were obtained with resolutions of 3.34 and 3.33 Å (Supplementary Fig. 5 and Table 1).

All resolutions were estimated by applying a soft mask around the protein density, and the gold-standard FSC = 0.413 criterion. ResMap was used to calculate the local resolution map.

### Model building, refinement, and validation

A de-novo atomic model was built in Coot,^47^ and amino acid assignment was achieved mainly based on the densities for bulky residues. Phenix^48^ was used to refine the model against the electron microscopy density map. Iterative cycles of refinement and manual rebuilding were carried out with phenix and refmac^49^ with secondary structure restraints, respectively. The models were validated using previously described methods, and figures were prepared using UCSF Chimera^50^ and PyMOL (http://www.pymol.org/).

### Data analysis statistics

The K_M_ values and maximal ATPase activity were analyzed by Michaelis–Menten equation on GraphPad Prism. Given the goodness of fit of the data, the R square values were 0.9 for the full-length wild-type ATP K_M_, 0.90 for the truncation mutant devoid of the N-terminal 54 residues ATP K_M_, and 0.94 for the truncation mutant devoid of the N-terminal 54 residue C26:0-CoA K_M_.

## Supplementary information

Supplemental material

Dataset 0A

Supplemental Vedio

Dataset 1B

Dataset 2B

Dataset 3B

Dataset 4B

Dataset 5B

Dataset 0B

Dataset 1A

Dataset 2A

Dataset 3A

Dataset 4A

Dataset 5A

## Data Availability

The accession numbers for the data reported in this paper are PDB: 7×07, 7X0T, 7XEC, 7X0Z, and 7X1W and EMDB: EMDB-32919, EMDB-32924, EMDB-33155, EMDB-32930, and EMDB-32951. The corresponding author could be contacted for more information.
