# Supplementary material for: Structural insights into substrate recognition and translocation of human peroxisomal ABC transporter ALDP
Source: Signal Transduct Target Ther. 2023 Feb 22;8:74. doi: 10.1038/s41392-022-01280-9 (PMC9944889; doi:10.1038/s41392-022-01280-9)
Supplement: Supplementary file 1 — Supplemental material [file 41392_2022_1280_MOESM1_ESM.docx]

Supplementary Materials for

**Structural Insights into Substrate Recognition and Translocation of** **Human Peroxisomal ABC Transporter ALDP**

Chao Xiong^1,2^*, Li-Na Jia^1,2^*, Wei-Xi Xiong^1,2^*, Xin-Tong Wu^1,2^, Liu-Lin Xiong^4^, Ting-Hua Wang^4^, Dong Zhou^1,2^, Zhen Hong^1,2^**^‡^**, Zheng Liu^3^**^‡^**, Lin Tang^1,2­­^**^‡^**

Correspondence to Lin Tang, Email: [ltang_xray@163.com](mailto:ltang_xray@163.com)

**This PDF file includes:**

Supplementary Figures. S1 to S17, and table S1

Movie S1

**Fig. S1.**


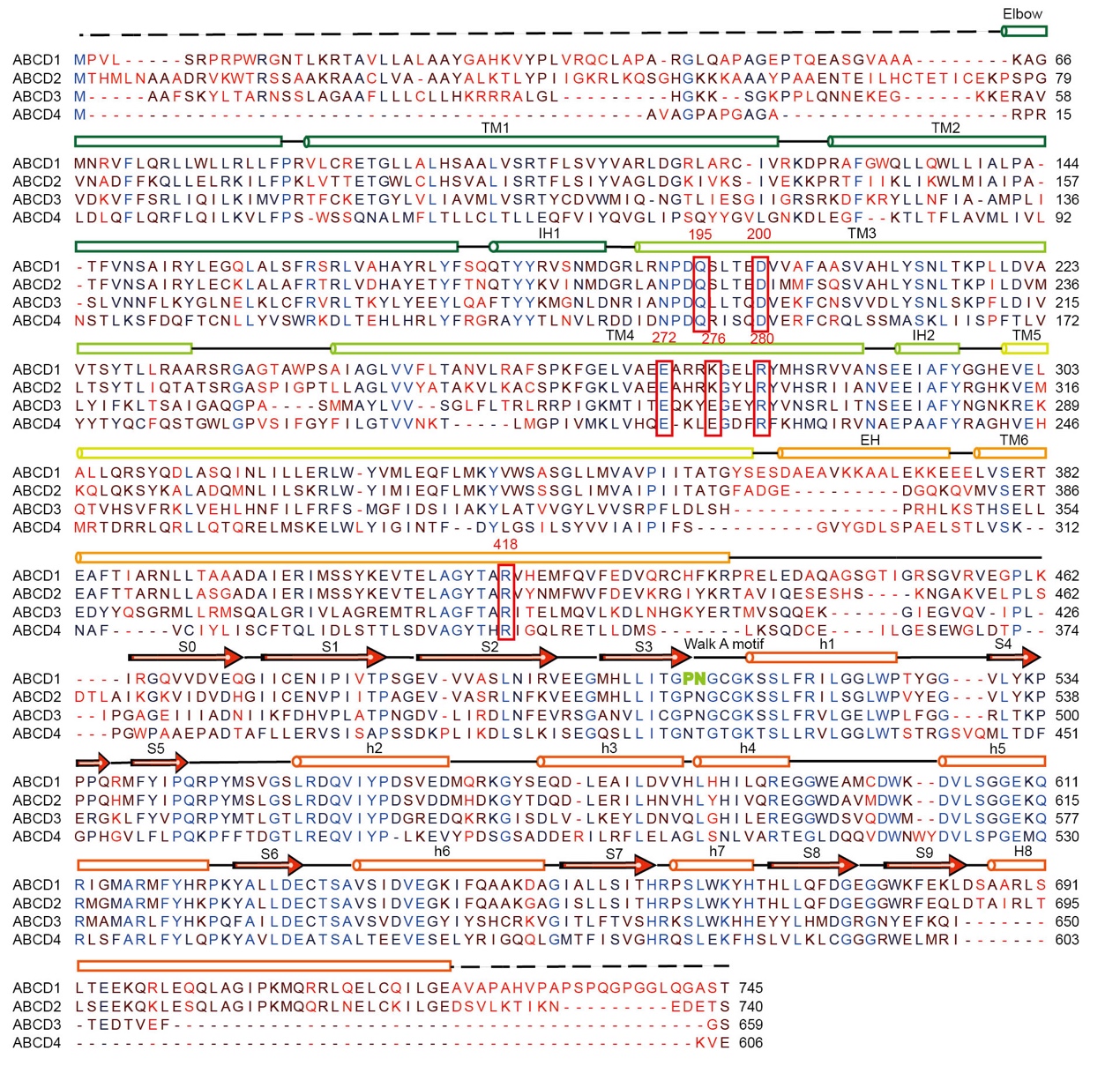


Supplementary Fig. 1 Sequence Alignment of Human ABCD1 and other Members of Human ABCD Family Transporters.

Secondary structure assignments are based on the human ABCD1 structure. Red squares mark the key charged residues at the inner surface of the vestibule of outward-facing ABCD1.

**Fig. S2.**


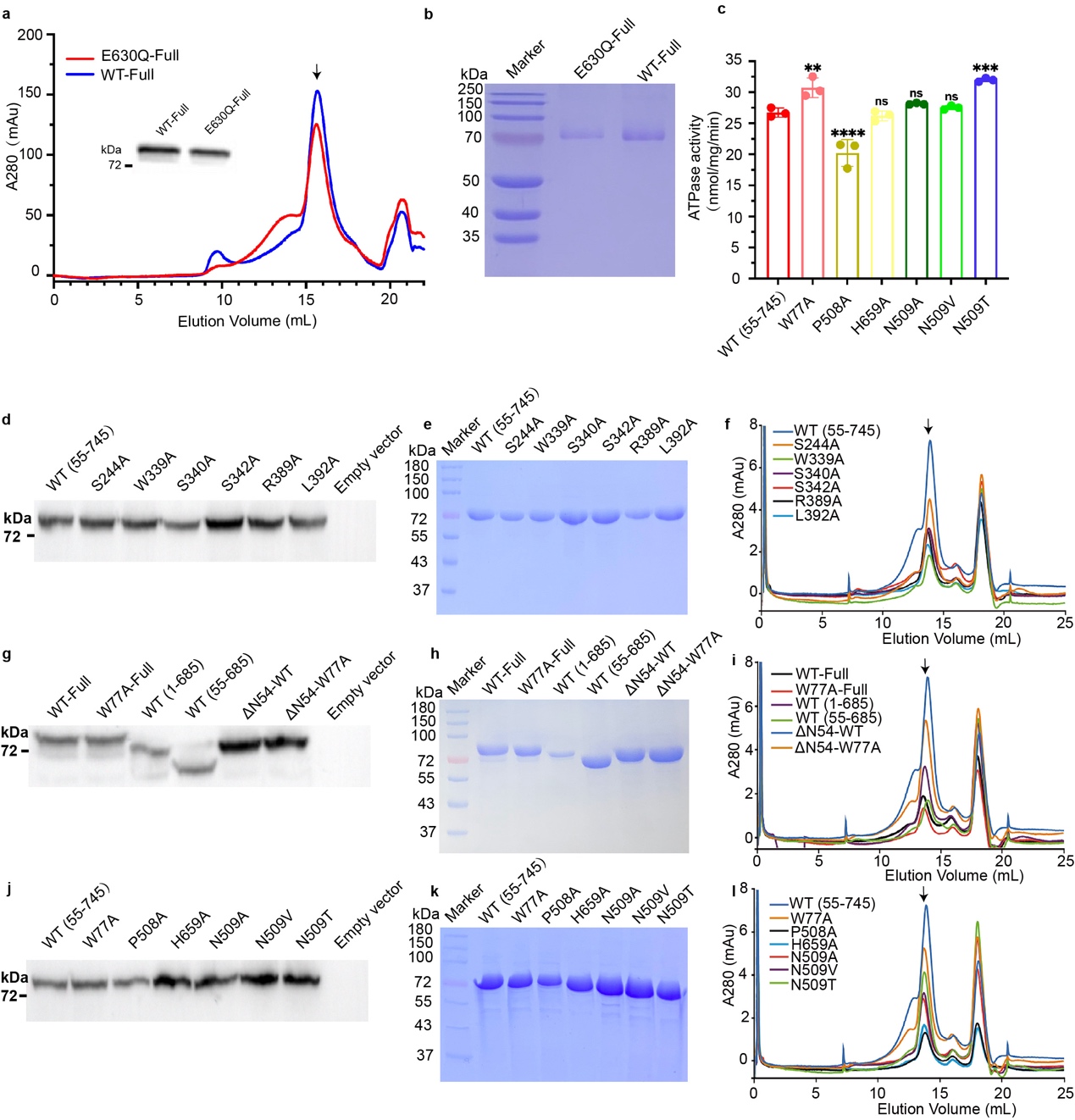


Supplementary Fig. 2 Biochemical Properties of Human ABCD1.

a-b. Size-exclusion profile, Western blot analysis and SDS-PAGE of purified wild-type and E630Q ABCD1.

c. ATPase activity of ABCD1 mutants. The reported errors represent standard deviation. ns = no significance，^✱✱✱✱^P < 0.0001, ^✱✱✱^P < 0.001, ^✱✱^P < 0.01, and ^✱^P < 0.05.

d, g and j, Western blot analysis of purified ABCD1 mutants using an anti-Flag Antibody.

e, h and k. SDS-PAGE of purified samples used for the ATPase activity assay.

f, i and l. Size-exclusion chromatography profile of purified ABCD1 mutants used for the ATPase activity assay. Arrows mark the fractions of ABCD1 constructs after running a Superpose 6 Increase SEC column.

**Fig. S3.**


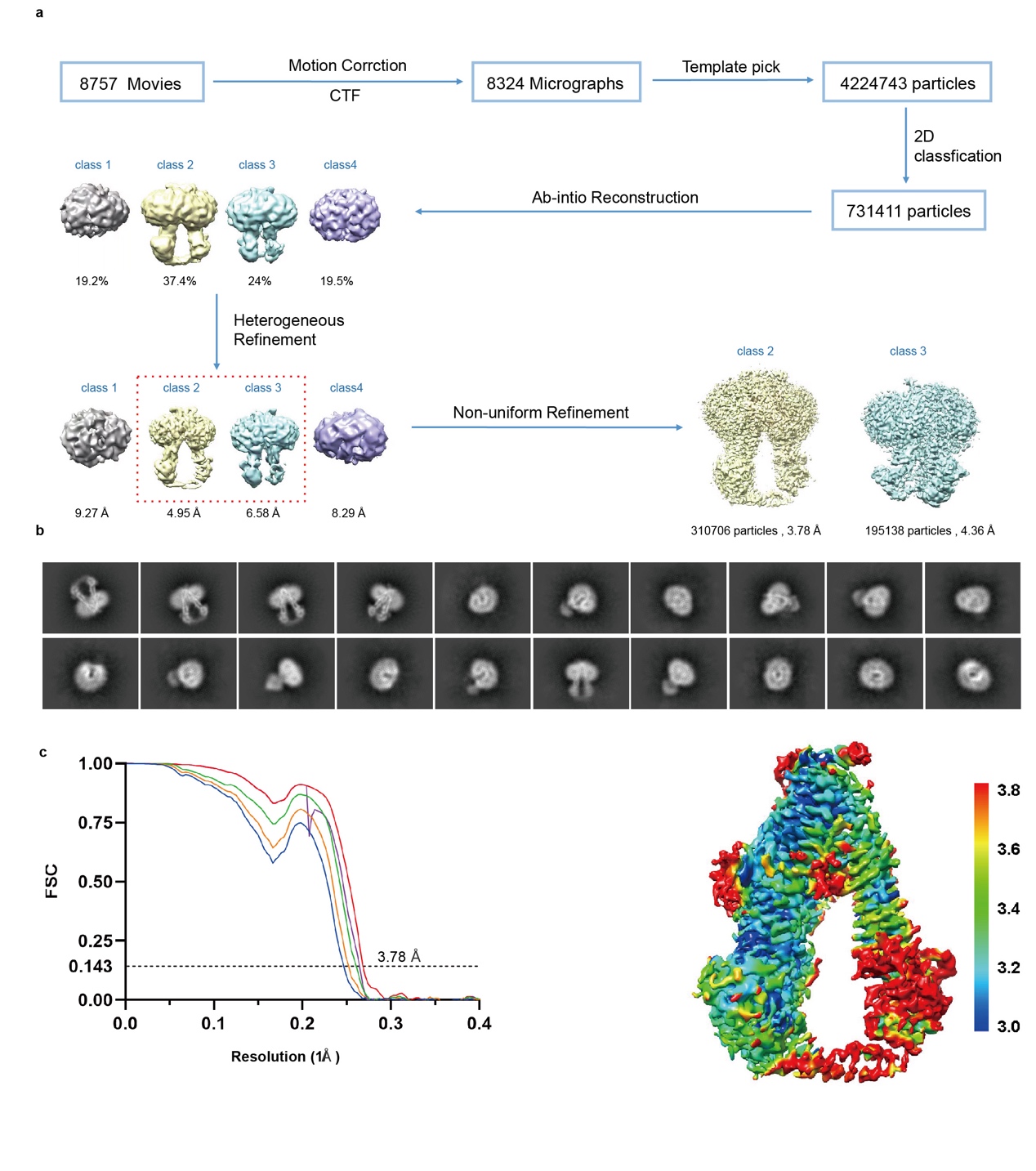


Supplementary Fig. 3 Structure Determination of ABCD1-WT-C26:0 Complex.

a. Flowchart of image processing for ABCD1-WT-C26:0 particles.

b. Representative 2D classes of ABCD1-WT-C26:0 complex.

c. Gold-standard FSC curves of the final 3D reconstructions and the density map colored by local resolution.

**Fig. S4.**


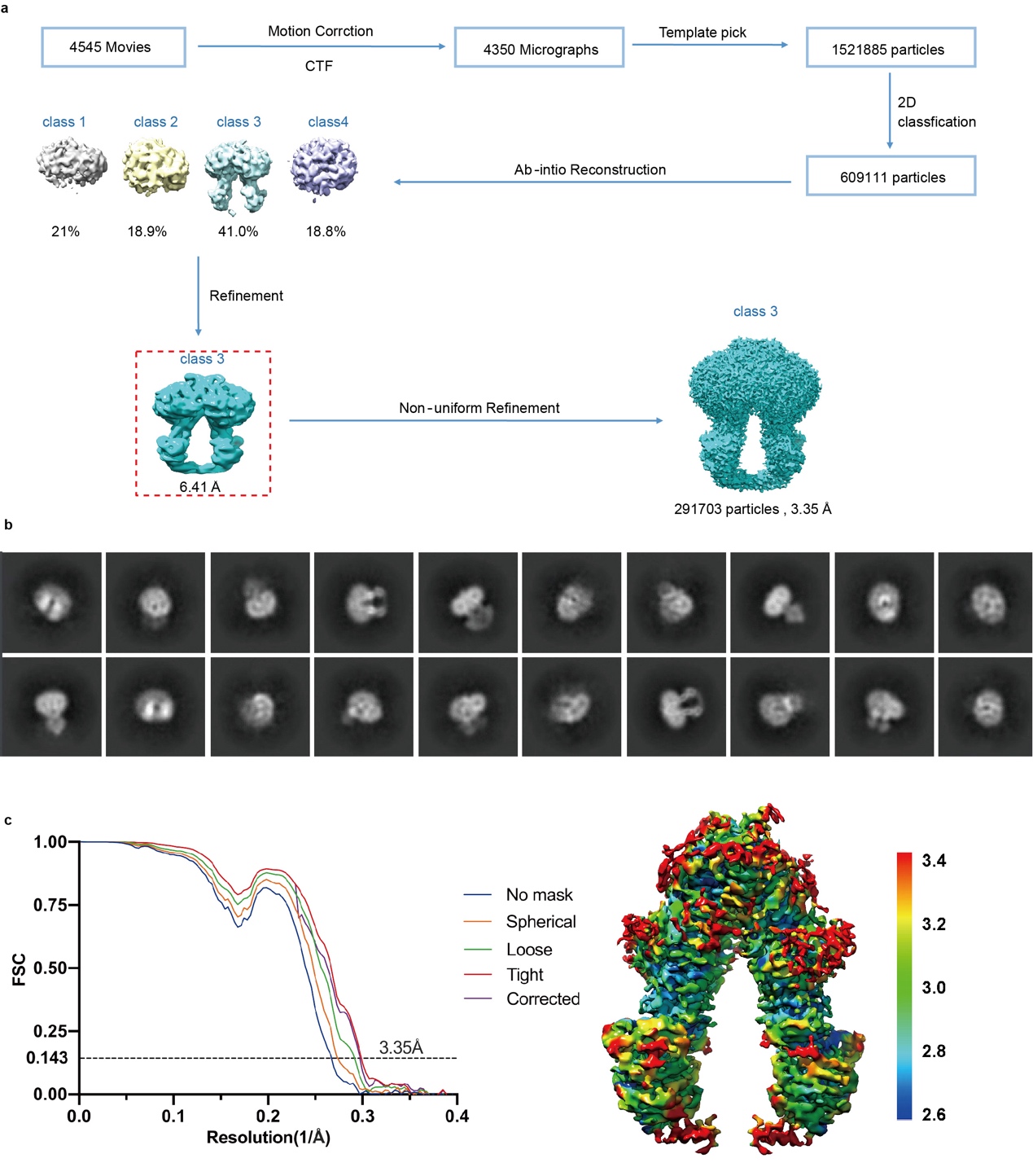


Supplementary Fig 4. Structure Determination of Apo ABCD1.

a. Flowchart of image processing for apo ABCD1 particles.

b. Representative 2D classes of apo ABCD1.

c. Gold-standard FSC curves of the final 3D reconstructions and the density map colored by local resolution.

**Fig. S5.**


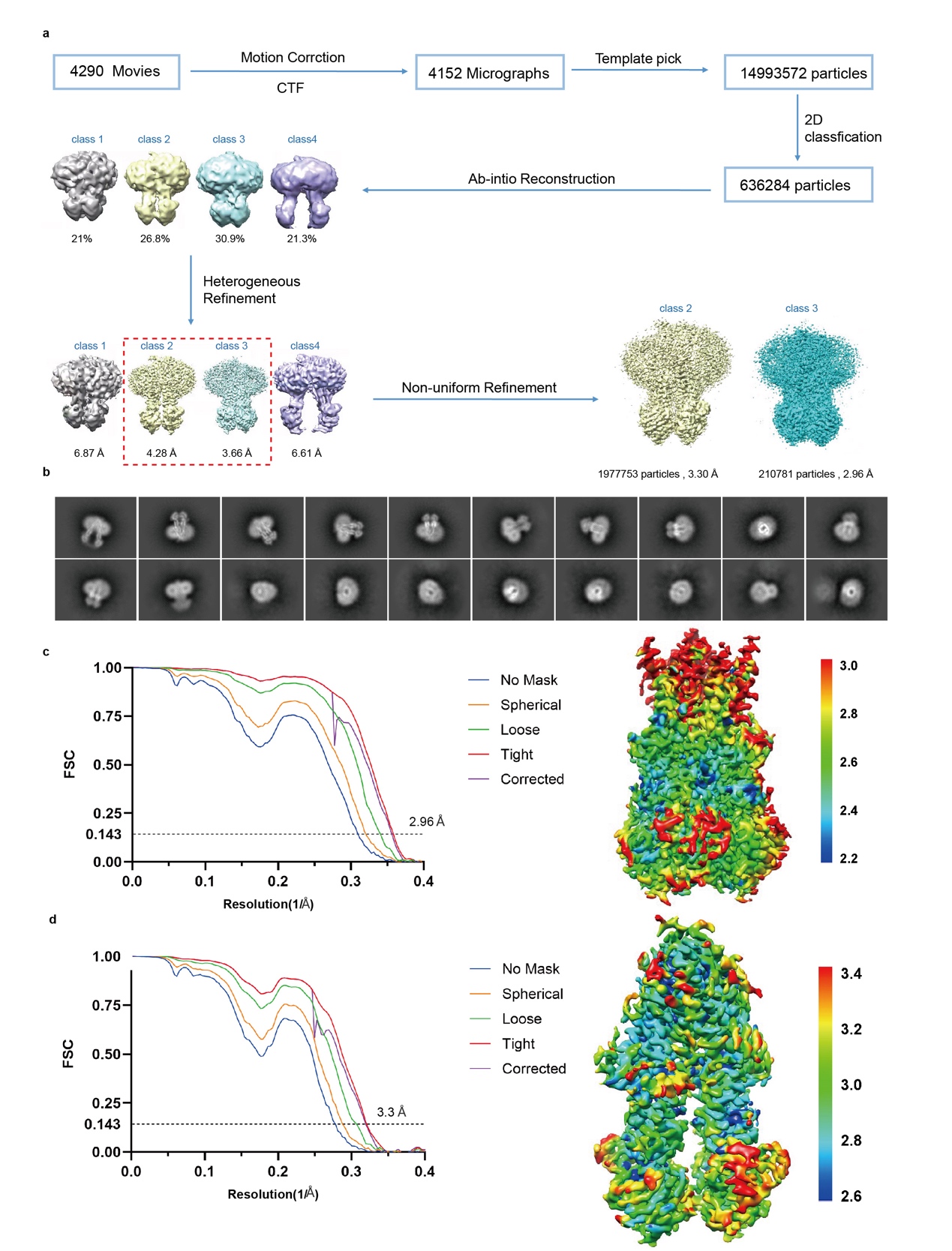


Supplementary Fig. 5 Structure Determination of ABCD1-E630Q-ATP Complex.

a. Flowchart of image processing for ABCD1-E630Q-ATP particles.

b. Representative 2D classes of ABCD1-E630Q-ATP complex.

c and d. Gold-standard FSC curves of the final 3D reconstructions and the density map colored by local resolution for the ABCD1-E630Q-ATP structure in outward-facing state and inward-facing state 3, respectively.

**Fig. S6.**


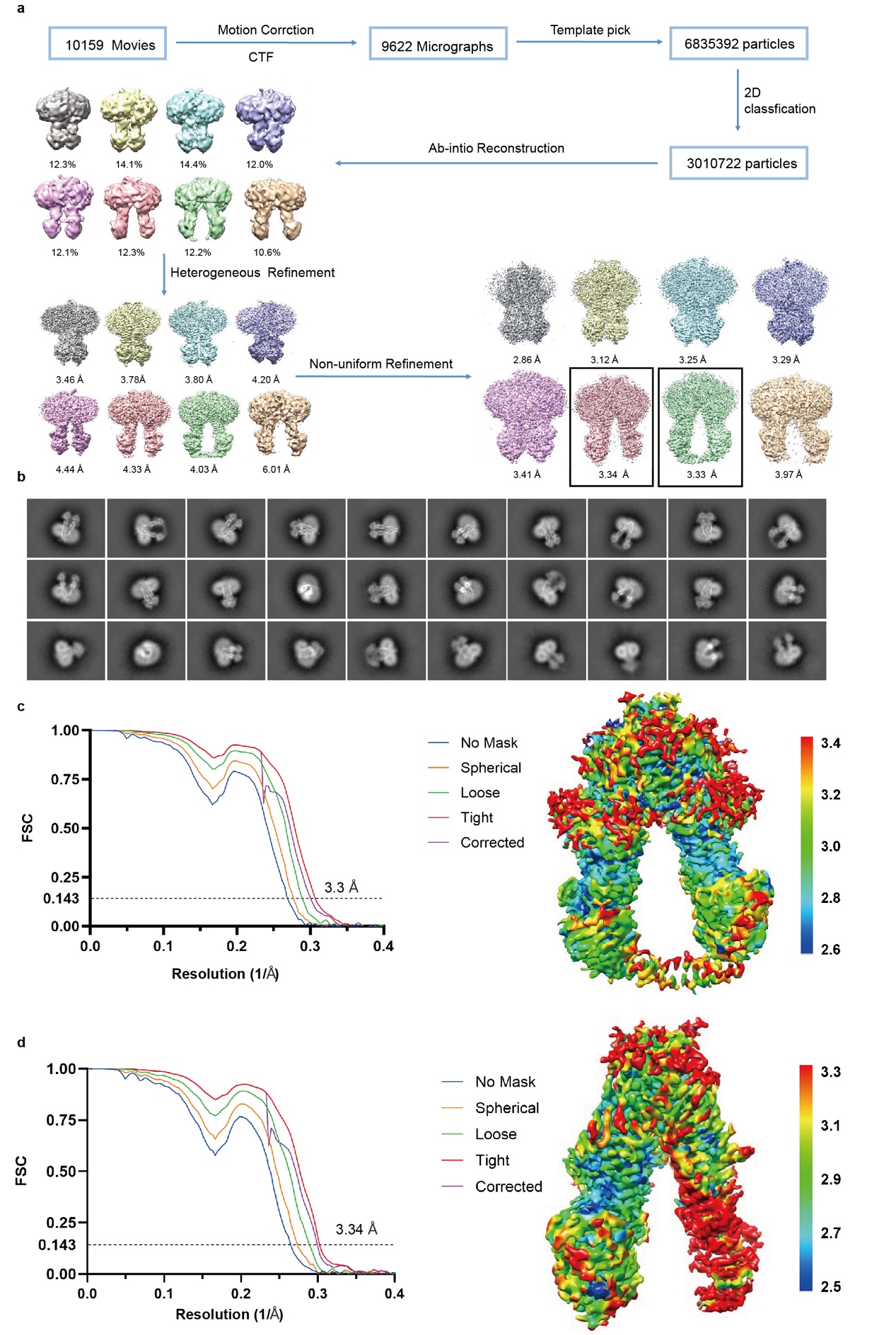


Supplementary Fig. 6 Structure Determination of ABCD1-E630Q in the Presence of C26:0-CoA and ATP.

a. Flowchart of image processing for the dataset collected on ABCD1-E630Q in the presence of C26:0-CoA and ATP.

b. Representative 2D classes.

c. Gold-standard FSC curves of the final 3D reconstructions and the density map colored by local resolution for ABCD1-E630Q-C26:0-CoA complex in inward-facing state 1.

d. Gold-standard FSC curves of the final 3D reconstructions and the density map colored by local resolution for ABCD1-E630Q-ATP complex in inward-facing state 2.

**Fig. S7.**


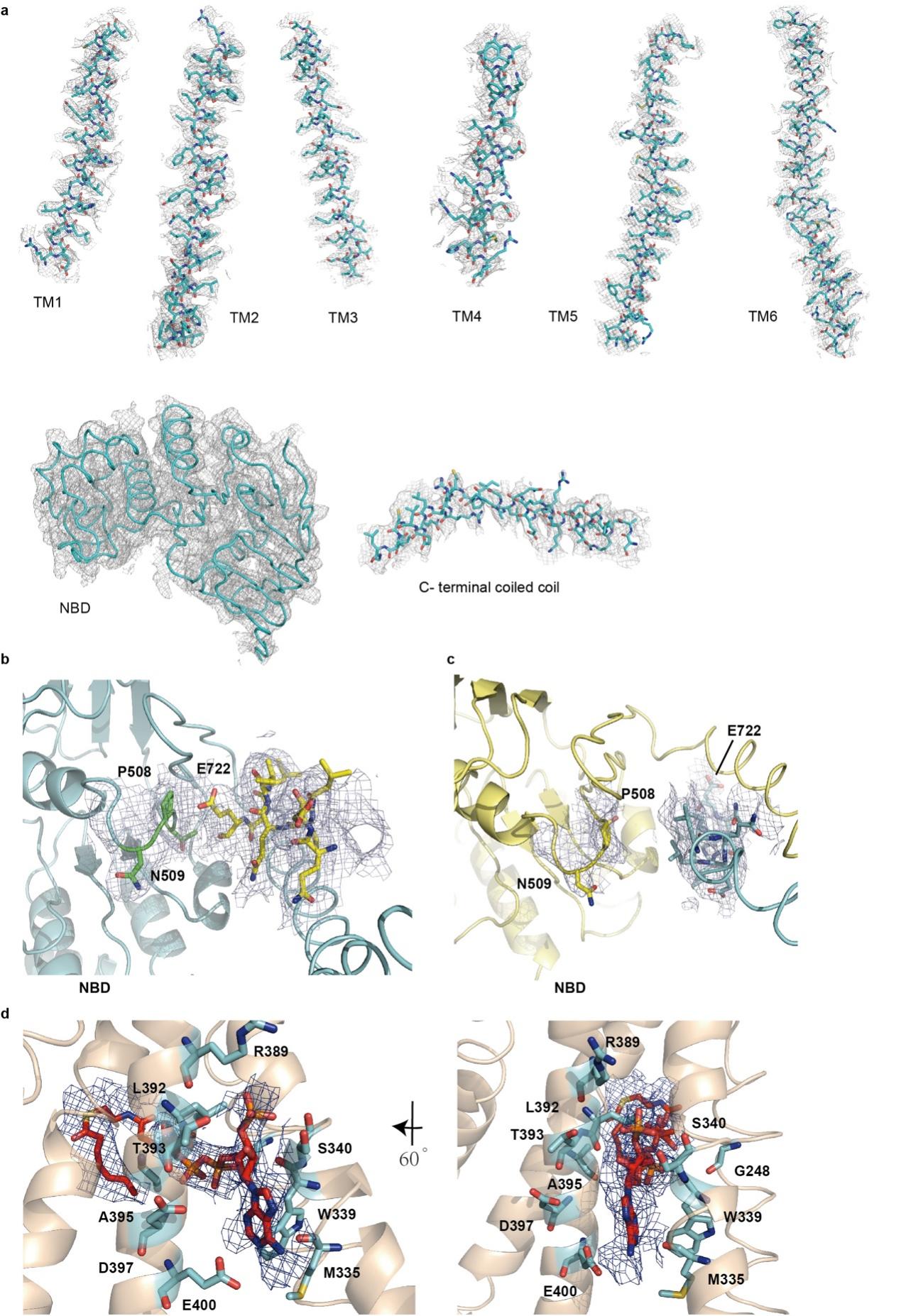


Supplementary Fig. 7 Quality of EM Density.

a. EM density of each transmembrane helix, the NBD, and the C-terminal coil.

b. Local structure of the C-terminal coiled coil of one NBD interacting with the Walker A motif of the other NBD.

c. Local structure of the C-terminal coiled coil of one subunit rotating away from the Walker A motif of the other NBD. The EM density maps in a-c are displayed at the same sigma level (4σ).

d. Stick representation of C26:0-CoA in different views. The EM density maps are displayed at the sigma level of (5σ).

**Fig. S8.**


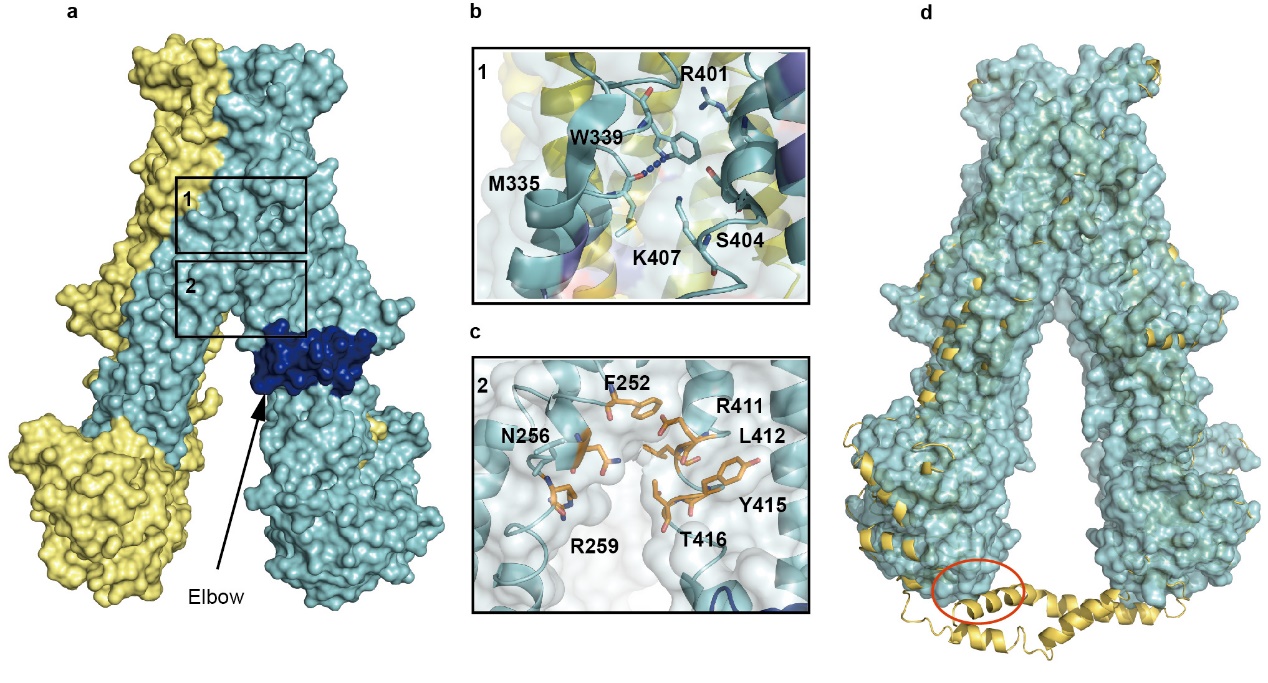


Supplementary Fig. 8 Inward-facing Intermediate Structure of ABCD1.

a. Surface representation of ABCD1 in inward-facing state 2.

b. Closed substrate-binding site near W339.

c. Potential modulation site near the elbow.

d. Structural comparison of inward-facing state 2 (surface representation in cyan) and inward-facing state 1 (cartoon representation in yellow orange) ABCD1 shows that the C terminal coiled coil of one subunit could sterically clash with the NBD domain of the other subunit.

**Fig. S9.**


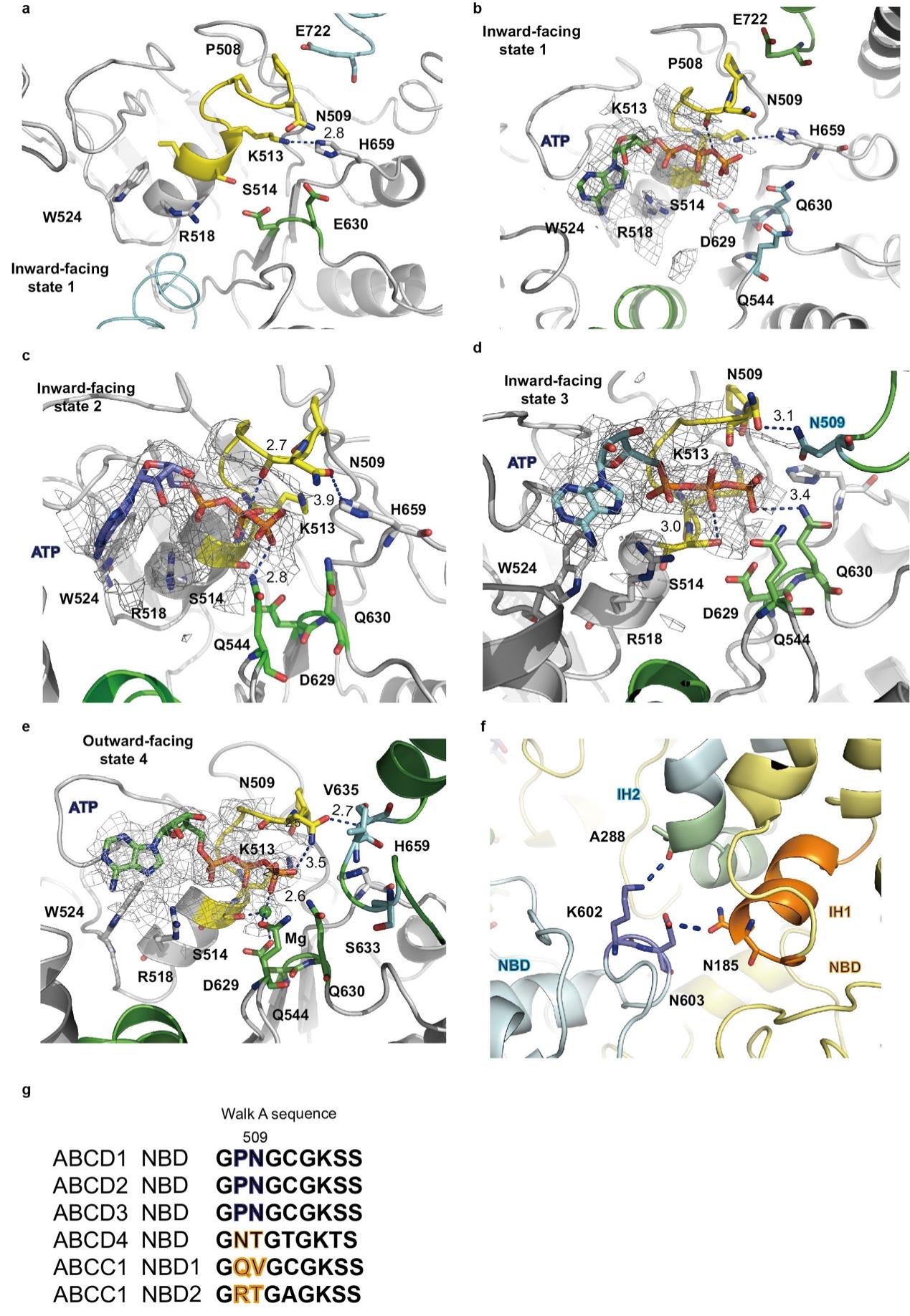


Supplementary Fig. 9 Structural Comparison of ATP Binding Site of ABCD1 in Different Conformational States.

a and b. Zoomed-in view of the ATP binding site of one NBD for ABCD1-WT-C26:0 complex, and ABCD1-E630Q-C26:0 complex.

c and d. Zoomed-in view of the ATP binding site of the ABCD1-E630Q-ATP in inward-facing states 2 and 3, respectively.

e. ATP molecule that interacts with Walker A motif and the signature motif of ABCD1-E630Q-ATP complex in outward-facing state.

f. Zoomed-in view of NBD–TMD interactions.

g. Sequence comparison of the Walker A motif between human ABCD1 and other ABC transporters. The EM density maps of ATP molecule are colored in grey and shown at the same sigma level (5σ).

**Fig. S10.**


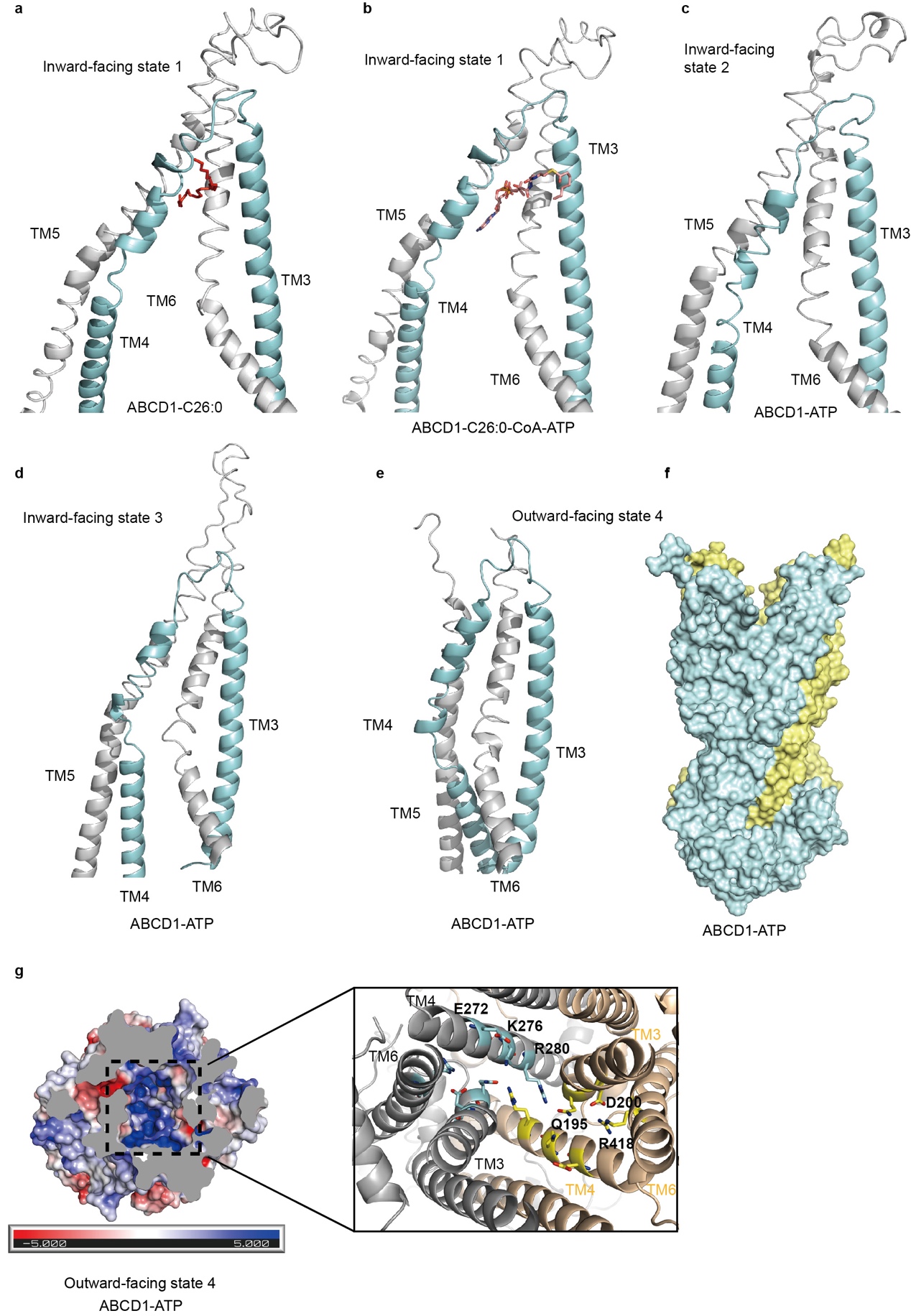


Supplementary Fig. 10 Structural Comparison of Four-helix Segments Between Different Conformational States.

a–e. Relative positions of four-helix TM4, TM3, TM5, TM6 of ABCD1 in different states.

f. Surface representation of ABCD1-ATP in outward-facing state.

g. Electrostatic property of the interior surface of outward-facing ABCD1. Conserved charged residues from TM3, TM4, TM6, and TM2 located at the bottom of the vestibule opening to the peroxisome lumen. Fig. S1 marks the residues with red square in sequence alignment.

**Fig. S11.**


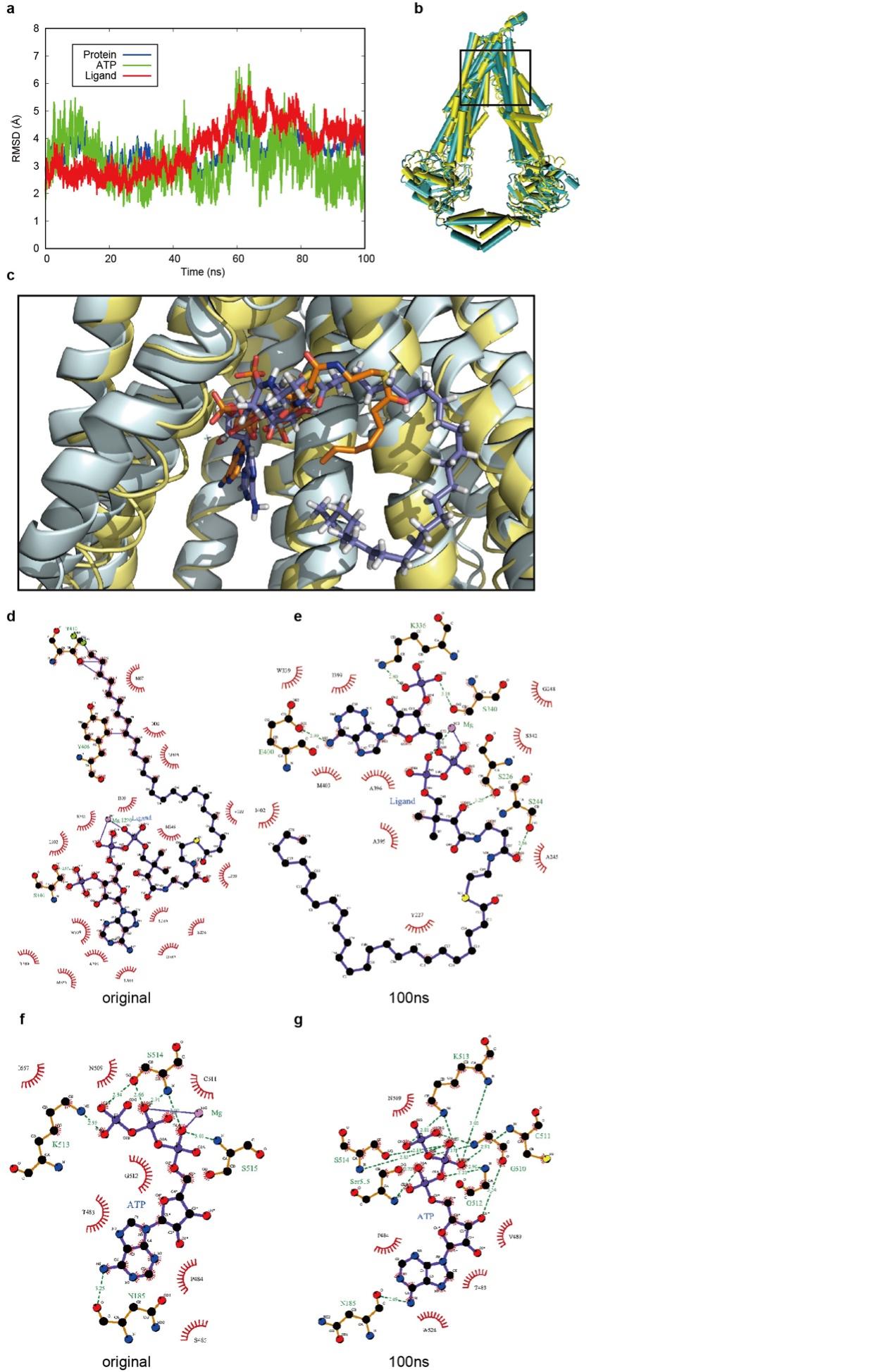


Supplementary Fig. 11 Molecular Dynamics Simulation of ABCD1-ATP-C26:0-CoA.

a. Root mean square deviation (RMSD) of the protein (blue), ATP (green) and the core region (excluding the aliphatic chain) of the ligand molecule (red) from the initial structure during MD simulation.

b. Overlay of the structures of the protein before (yellow) and after (cyan) the 100-ns simulation.

c. Zoomed-in view of the ligand binding site before and after molecular simulation.

d–e. Interaction between the ligand molecule and its surrounding in the initial structure and after the 100–ns simulation.

f–g. Interaction between the ATP molecule and its surrounding in the initial structure (left) and after the 100-ns simulation (right).

**Fig. S12.**


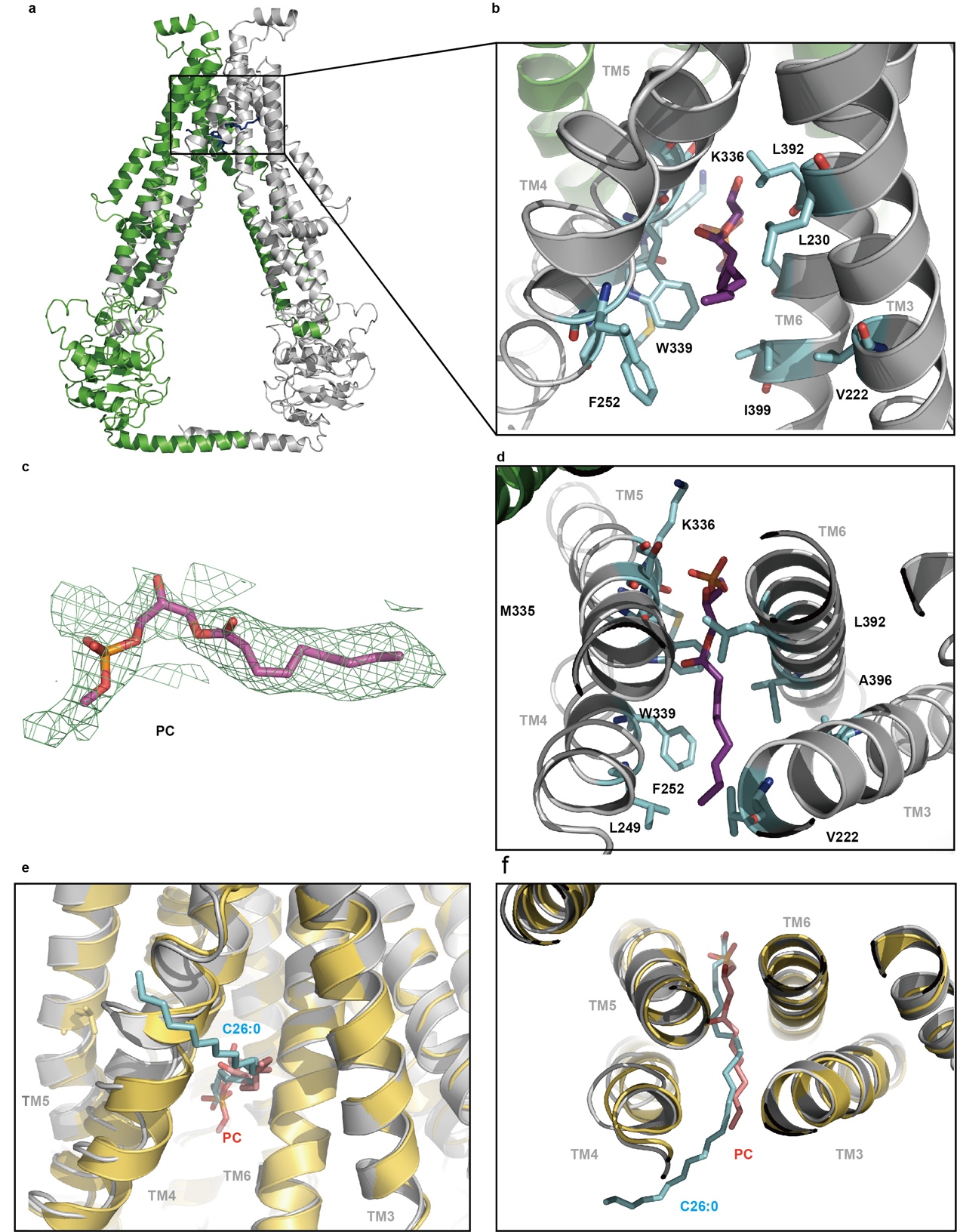


Supplementary Fig. 12 Molecular Architecture of Apo ABCD1.

a. Overall structure of ABCD1 in the absence of any ligand.

b and d. Zoomed-in views of the lipid binding site.

c. EM density of an endogenous membrane lipid. The EM density map is displayed at the sigma level of (5σ).

e–f. Superimposition of the apo (yellow) and C26:0-bound (gray) structures.

**Fig. S13.**


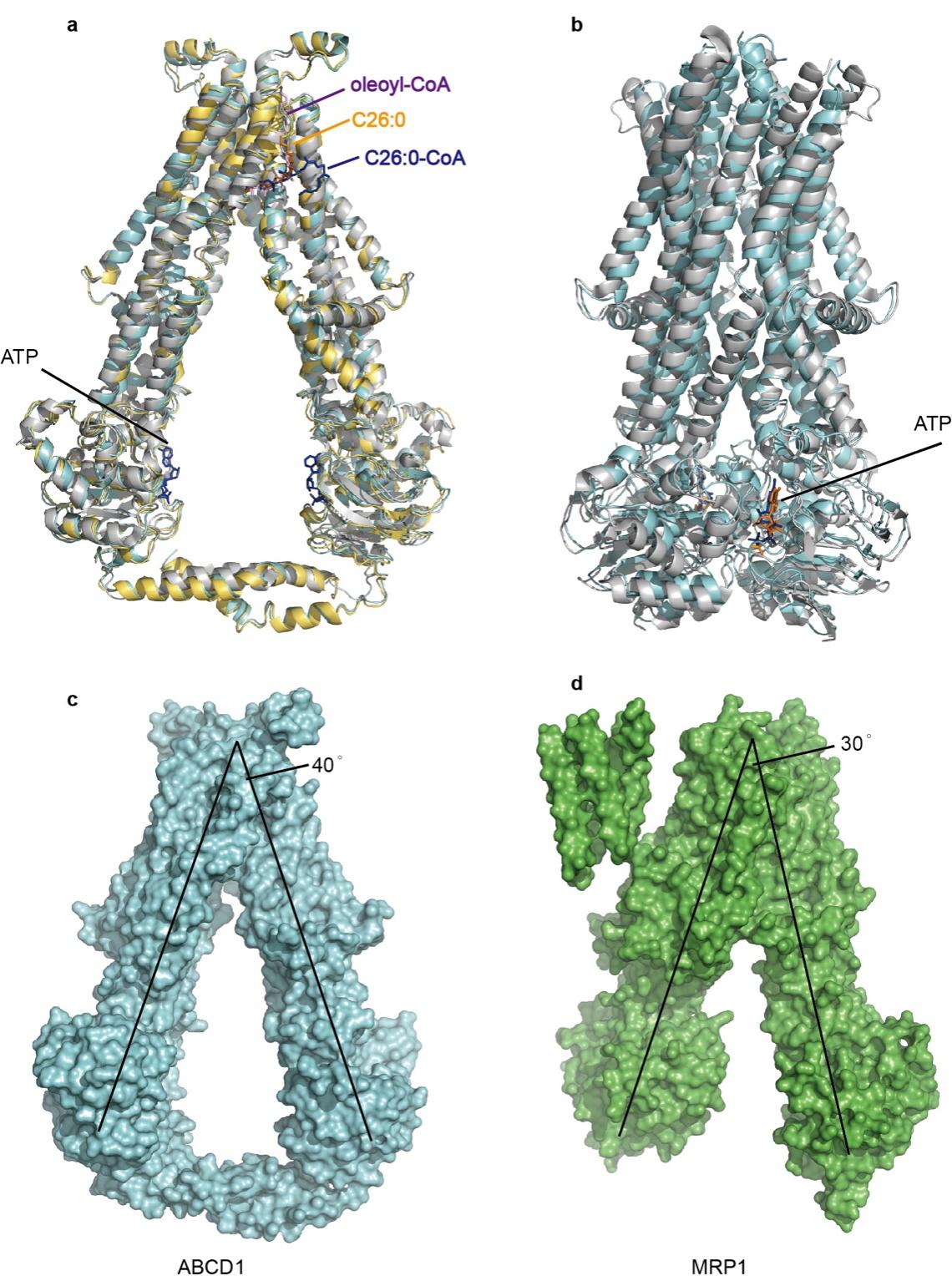


Supplementary Fig. 13 Comparison of ABCD1 Structures in Different Conformational States.

a. A structural comparison among ABCD1-C26:0-CoA (cyan), ABCD1-C26:0 (yellow) and ABCD1-oleoyl-CoA (grey, PDB ID:7SHN) shows their overall structures are highly similar.

b. Structural comparison of ABCD1-ATP structures in outward conformational states. The structure of ATP-bound ABCD1 determined in this study is colored in cyan, and the ABCD1-ATP structure (PDB ID: 7RRA) reported by Alam et al. is shown in grey.

c-d. Structural comparison between ABCD1 and MRP1 (PDB ID:5UJ9) indicates that the angle between the two TMD bundles of ABCD1 is higher than that of MRP1.

**Fig. S14.**


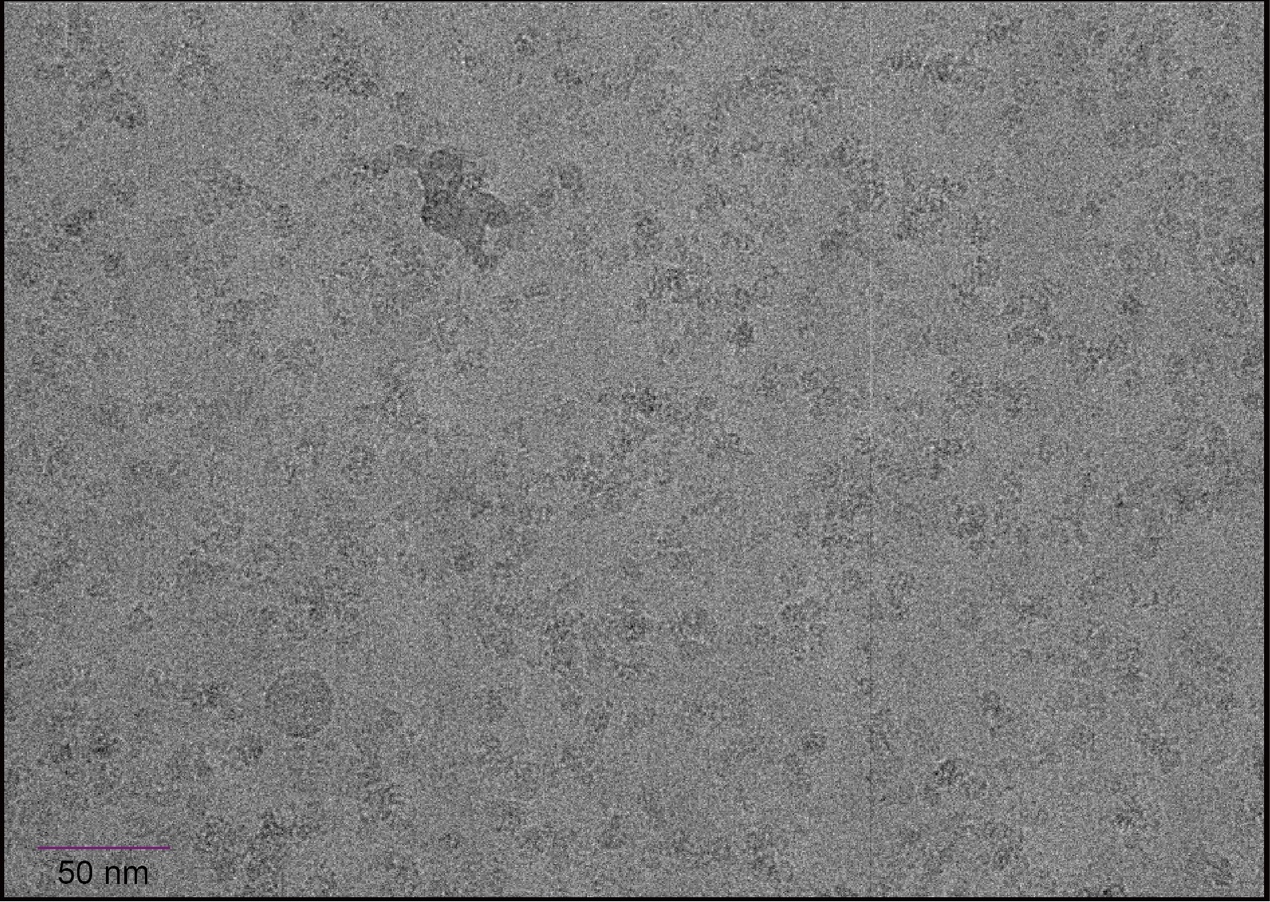


Supplementary Fig. 14 Representative Cryo-EM Micrograph of ABCD1-WT in the Presence of C26:0.

**Fig. S15.**

**
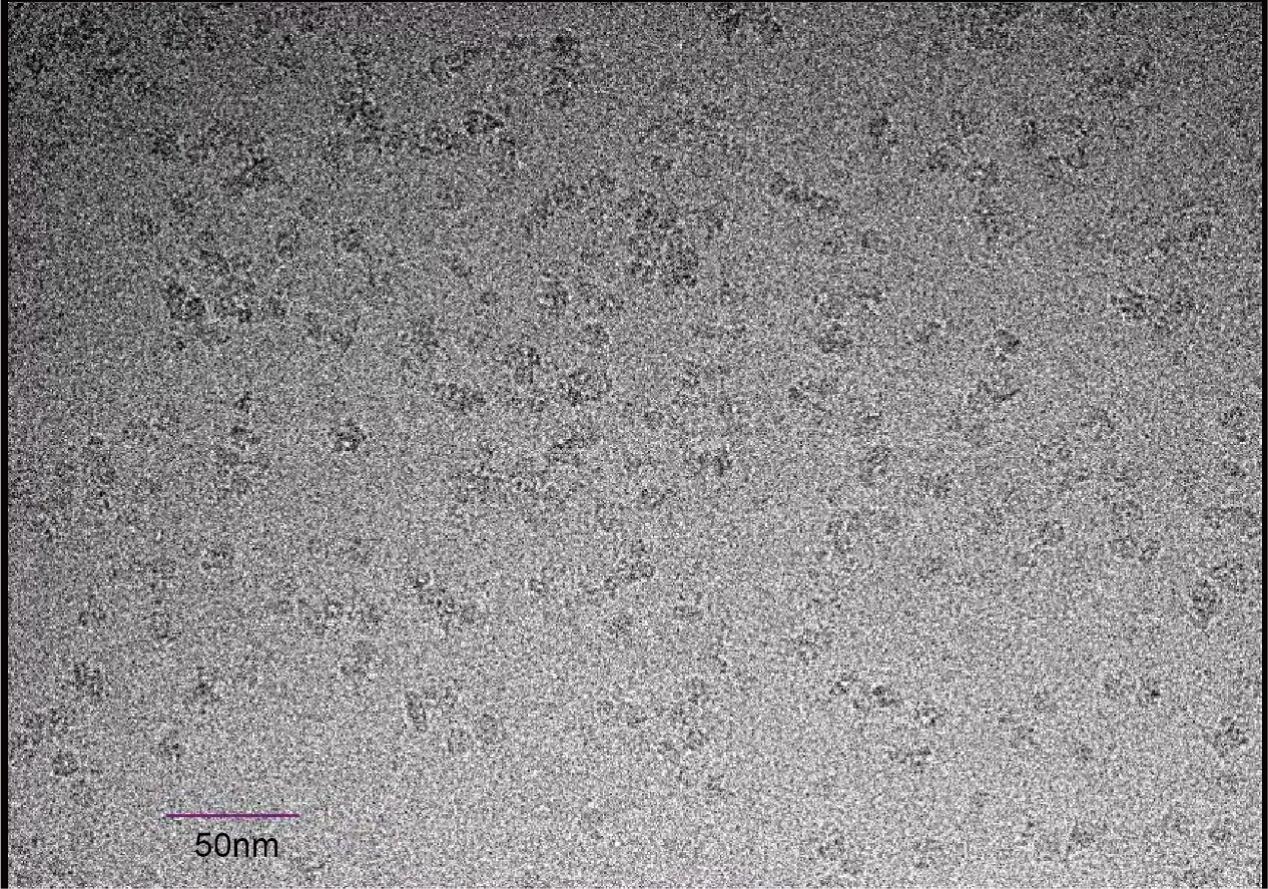
**

Supplementary Fig. 15 Representative Cryo-EM Micrograph of apo ABCD1.

**Fig. S16.**


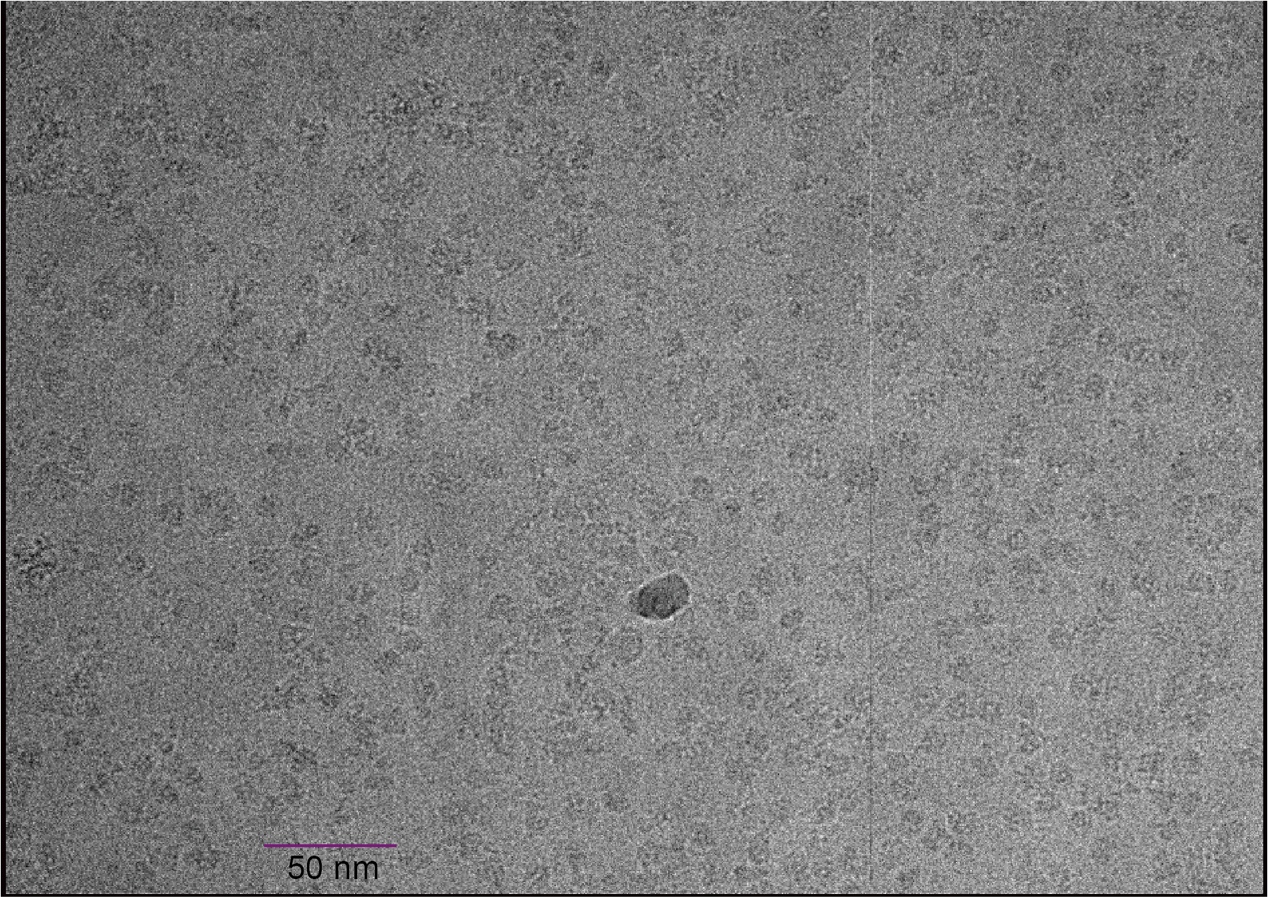


Supplementary Fig. 16 Representative Cryo-EM Micrograph of ABCD1-E630Q in the Presence of C26:0-CoA and ATP.

**Fig. S17.**


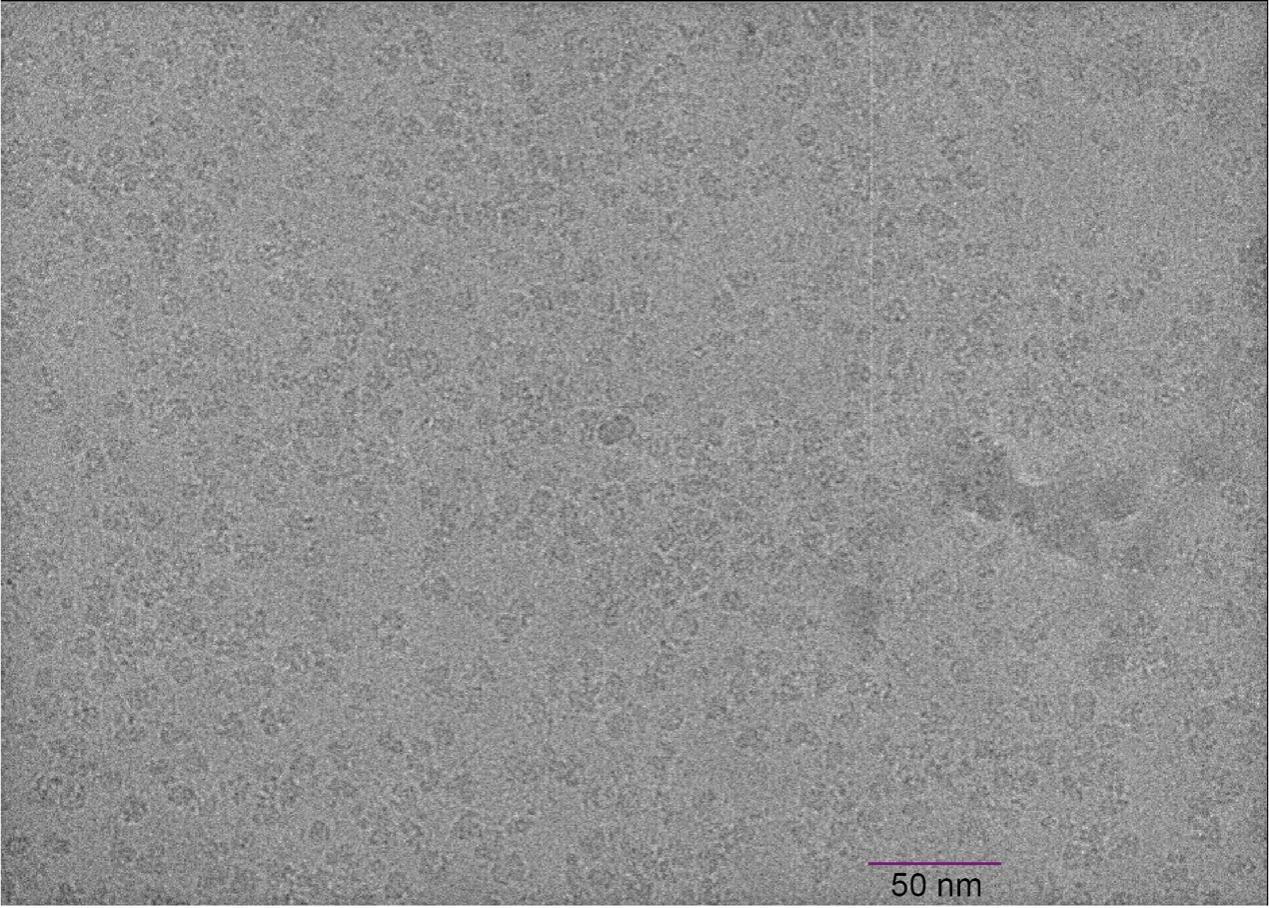


Supplementary Fig. 17 Representative Cryo-EM Micrograph of ABCD1-E630Q in the Presence of ATP.

**Table S1.**

Cryo-EM data collection, refinement and validation statistics

|  | #0 ABCD1-WT（EMDB-34064）（PDB 7YRQ） | #1 ABCD1-WT-C26:0  (EMDB-32919)  (PDB 7X07) | #2 ABCD1-E630Q-C26:0-COA-ATP  (EMDB-32924)  (PDB 7X0T) | #3ABCD1-E630Q-ATP  (EMDB-33155)  (PDB 7XEC) | #4ABCD1-E630Q-ATP  (EMDB-32930)  (PDB 7X0Z) | #5 ABCD1-E630Q-ATP  (EMDB-32951)  (PDB 7X1W) |
| --- | --- | --- | --- | --- | --- | --- |
| **Data collection and processing** |  |  |  |  |  |  |
| Magnification | 105K | 105K | 105K | 105K | 105K | 105K |
| Voltage (kV) | 300 | 300 | 300 | 300 | 300 | 300 |
| Electron exposure (e–/Å^2^) | 55 | 66.5 | 66.5 | 66.5 | 66.5 | 66.5 |
| Defocus range (μm) | -1.5 to -2.5 | -1.5 to -2.5 | -1.5 to -2.5 | -1.5 to -2.5 | -1.5 to -2.5 | -1.5 to -2.5 |
| Pixel size (Å) | 0.819 | 0.83 | 0.83 | 0.83 | 0.83 | 0.83 |
| Symmetry imposed | C2 | C1 | C1 | C1 | C1 | C1 |
| Initial particle images (no.) | 1521885 | 4224743 | 6835392 | 6835392 | 14993572 | 14993572 |
| Final particle images (no.) | 291703 | 731411 | 3010722 | 3010722 | 636284 | 636284 |
| Map resolution (Å)  FSC threshold | 0.143 | 0.143 | 0.143 | 0.143 | 0.143 | 0.143 |
| Map resolution range (Å) | 3.35 | 3.78 | 3.30 | 3.34 | 2.96 | 3.30 |
|  |  |  |  |  |  |  |
| **Refinement** |  |  |  |  |  |  |
| Initial model used (PDB code) |  |  |  |  |  |  |
| Model resolution (Å)  FSC threshold | 0.143 | 0.143 | 0.143 | 0.143 | 0.143 | 0.143 |
| Model resolution range (Å) | 3.35 | 3.78 | 3.3 | 3.34 | 2.96 | 3.30 |
| Map sharpening *B* factor (Å^2^) | 144.5 | 141.1 | 132.6 | 126.9 | 87.6 | 111.5 |
| Model composition  Non-hydrogen atoms  Protein residues  Ligands | 9814  1247  2 | 10129  1267  1 | 10239  1267  2 | 9507  1188  1 | 9258  1156  2 | 9317  1159  2 |
| *B* factors (Å^2^)  Protein  Ligand | 60.3  57.4 | 67.39  46.04 | 91.01  141.71 | 69.16  57.83 | 41.12  32.25 | 40.21  62.94 |
| R.m.s. deviations  Bond lengths (Å)  Bond angles (°) | 0.007  1.262(10) | 0.013 (7)  1.660 (90) | 0.008 (2)  1.296 (52) | 0.008 (0)  1.436 (27) | 0.005 (0)  0.606 (1) | 0.005 (0)  0.632 (2) |
| Validation  MolProbity score  Clashscore  Poor rotamers (%) | 2.20  4.16  5.20 | 2.18  8.10  1.97 | 2.15  6.85  1.97 | 2.24  5.30  3.61 | 1.79  6.87  0.00 | 1.93  7.97  0.00 |
| Ramachandran plot  Favored (%)  Allowed (%)  Disallowed (%) | 92.74  7.02  0.24 | 91.18  8.11  0.72 | 89.83  10.02  0.16 | 90.75  9.08  0.17 | 93.88  6.12  0.00 | 91.63  8.28  0.09 |

**Movie S1.**

Conformational changes of ABCD1 from the inward-facing state 1 to the outward-facing state 4. In the absence of C26:0-COA and ATP, ABCD1 adopts a wide-open, inward-facing structure (inward-facing state 1). In the presence of C26:0-COA and ATP, the transmembrane region and the nucleotide-binding domains (NBDs) undergo concerted conformational movements. Accordingly, ABCD1 transitions from the inward-facing state 1 to the outward-facing state 4 through the intermediate inward-facing states (inward-facing state 2 and inward-facing state 3) and resets the transport cycle.
